# Gene profile of fibroblasts identify relation of CCL8 with idiopathic pulmonary fibrosis

**DOI:** 10.1186/s12931-016-0493-6

**Published:** 2017-01-05

**Authors:** Jong-Uk Lee, Hyun Sub Cheong, Eun-Young Shim, Da-Jeong Bae, Hun Soo Chang, Soo-Taek Uh, Young Hoon Kim, Jong-Sook Park, Bora Lee, Hyoung Doo Shin, Choon-Sik Park

**Affiliations:** 1 Department of Interdisciplinary Program in Biomedical Science Major, Soonchunhyang Graduate School, Bucheon, Korea; 2 Department of Genetic Epidemiology, SNP Genetics, Inc., Sogang University, Seoul, Korea; 3 Department of Life Science, Sogang University, Seoul, Korea; 4 Division of Allergy and Respiratory Medicine, Department of Internal Medicine, Soonchunhyang, University Bucheon Hospital, Bucheon, Korea; 5 Division of Respiratory and Allergy Medicine, Department of Internal Medicine, Soonchunhyang University Seoul Hospital, 1174, Jung Dong, Wonmi-Gu, Bucheon, Gyeonggi Do 420-021 Korea; 6 Division of Respiratory Medicine, Department of Internal Medicine, Soonchunhyang University, Chunan Hospital, Cheonan, Korea; 7 Department of Biostatistic Consulting, Soon Chun Hyang Medical Center, Bucheon, Korea

**Keywords:** Gene expression, IPF, CCL8, Transcriptome

## Abstract

**Background:**

Idiopathic pulmonary fibrosis (IPF) is characterized by the complex interaction of cells involved in chronic inflammation and fibrosis. Global gene expression of a homogenous cell population will identify novel candidate genes.

**Methods:**

Gene expression of fibroblasts derived from lung tissues (8 IPF and 4 controls) was profiled, and ontology and functional pathway were analyzed in the genes exhibiting >2 absolute fold changes with *p*-values < 0.05. *CCL8* mRNA and protein levels were quantified using real-time PCR and ELISA. *CCL8* localization was evaluated by immunofluorescence staining.

**Results:**

One hundred seventy eight genes differentially expressed and 15 genes exhibited >10-fold change. Among them, 13 were novel in relation with IPF. *CCL8* expression was 22.8-fold higher in IPF fibroblasts. The levels of *CCL8* mRNA and protein were 3 and 9-fold higher in 14 IPF fibroblasts than those in 10 control fibroblasts by real-time PCR and ELISA (*p* = 0.022 and *p* = 0.026, respectively). The *CCL8* concentrations in BAL fluid was significantly higher in 86 patients with IPF than those in 41 controls, and other interstitial lung diseases including non-specific interstitial pneumonia (*n* = 22), hypersensitivity pneumonitis (*n* = 20) and sarcoidosis (*n* = 19) (*p* < 0.005, respectively). Cut-off values of 2.29 pg/mL and 0.43 pg/mL possessed 80.2 and 70.7% accuracy for the discrimination of IPF from NC and the other lung diseases, respectively. IPF subjects with *CCL8* levels >28.61 pg/mL showed shorter survival compared to those with lower levels (*p* = 0.012). *CCL8* was expressed by α-SMA-positive cells in the interstitium of IPF.

**Conclusions:**

Transcriptome analysis identified several novel IPF-related genes. Among them, *CCL8* is a candidate molecule for the differential diagnosis and prediction of survival.

**Electronic supplementary material:**

The online version of this article (doi:10.1186/s12931-016-0493-6) contains supplementary material, which is available to authorized users.

## Background

Idiopathic pulmonary fibrosis (IPF) is characterized by alveolar epithelial cell hyperplasia and increased myofibroblast with the interstitial deposition of extracellular matrix (ECM) [1]. The disease course is highly variable due to interactions between chronic inflammatory and fibrosis-related processes [2, 3]. Exploration of global gene expression in lung tissues may facilitate the identification of novel candidate genes to further explain the complex mechanism and to predict the clinical courses of IPF. In a human study, 164 differentially expressed genes were demonstrated in IPF lung tissues [4]. In this study, fibrotic lungs showed changes in the expression of genes involved in ECM formation and degradation. A comparison of rapid-and slow-progressor patients revealed 437 differentially expressed genes involved in morphogenesis [5]. In another study, integration of the expression levels of 134 genes enabled the discrimination of progressive and stable subjects [6]. Almost identical patterns of gene expression to those of stable IPF were reported in cases of acute exacerbation [7]. These studies have demonstrated novel candidate genes related with IPF.

The use of whole-lung tissues, however, may be a limitation of transcriptomic studies because transcriptomic changes are cell-type-specific [8]. The pathologic characteristics of IPF include mixed features with normal lungs, alveolar inflammation, interstitial fibrosis, and honeycomb changes [2]. Furthermore, the extent of fibrosis and inflammation varies markedly during the disease course. Accordingly, selective separation of homogenous cells from diseased lungs would be optimal, but is problematic. Among the various cell types present in lung tissue, fibroblasts are easily obtained and maintained, and the biologic properties of IPF fibroblasts differ from those of normal lung fibroblasts [9, 10]. To further investigate the molecular mechanisms of IPF lungs, a global transcriptome analysis was conducted using fibroblasts obtained from the lung tissues of 8 patients with IPF and normal lungs of 4 subjects with localized lung lesions. The differential expression of CCL8 was validated using an additional number of fibroblasts and bronchoalveolar lavage (BAL) fluid samples from normal controls (NC), patients with IPF and those with other interstitial lung diseases including non-specific interstitial pneumonia (NSIP), hypersensitivity pneumonitis (HP), and sarcoidosis.

## Methods

### Study subjects

Plasma, BAL fluids, and lung tissues of the subjects with diffuse interstitial lung disease were obtained from a biobank in Soonchunhyang University Bucheon Hospital (schbc-biobank-2014-005-1, schbc-biobank-2014-005-2) after the study protocol was approved by institutional review board (IRB) in Korea National Institute for Bioethics Policy (KoNIBP; P01-201408-BS-01-00). Control BAL fluids were obtained from general population and hospital personnel, and blood samples were obtained from spouses, general population and hospital personnel after approval by the hospital ethics committee (SCHBC 2015-08-025-005, schbc-biobank-2015-013). An informed written consent to participate was obtained from each subject. The diagnostic criteria for IPF, NSIP, HP and sarcoidosis were based on the international consensus statement [1, 11–14]. All subjects were examined by physicians to obtain their medical history and underwent a chest X-ray, pulmonary function tests, high-resolution chest computed tomography (HRCT), and blood tests to exclude collagen vascular diseases. None of the IPF patients had any evidence of underlying collagen vascular diseases through clinical manifestations or laboratory tests. IPF was diagnosed by the presence of a UIP pattern in the pathological specimen (surgical IPF) and/or by HRCT in patients who were not subjected to surgical lung biopsy (clinical IPF). Two pathologists examined each slide independently after they were informed of the patients’ age, sex, and HCRT results. The pathologic recognition of the NSIP pattern included two major aspects: (1) recognition of the characteristic histologic features and (2) exclusion of other patterns of ILD as described in the ATS/ERS 2002 classification [14, 15], previous publication of ours [16] and the modified version on the histologic definition of the NSIP pattern [17].

HP was diagnosed by the presence of compatible clinical manifestations with a non-necrotizing granulomatous interstitial bronchiolocentric pneumonitis [11]. The diagnosis of sarcoidosis was made on the basis of the compatible clinical pictures and histologic demonstration of noncaseating granulomas [12, 13] The diagnosis of HP and sarcoidosis needed exclusion of other diseases capable of producing a similar histologic picture: Biopsy tissues were subjected to special stains (acid fast bacilli stain and Gömöri methenamine silver stain) to rule out microorganisms and fungi. IPF patients were evaluated using serial FVC and DLCO measurements. The annual rate of FVC decline [dFVC(%/year)] was calculated as follows: (last FVC - baseline FVC)/baseline FVC/year. The normal controls had no respiratory symptoms, as determined by a screening questionnaire [18], had a predicted FEV1 and FVC > 80%, and had normal chest radiogram results.

### Fibroblast culture

Lung fibroblasts were cultured from the surgical specimens of 14 patients with IPF and normal lungs of 10 subjects who underwent surgery to remove stage I or II lung cancer as described in the previous publication [19]. Briefly, lung specimens were finely minced and placed into 150 cm^2^ cell culture flasks with tissue culture media (TCM) consisting of DMEM (Lonza Walkersville, Inc., Walkersville, MD, USA), 10% fetal bovine serum (Thermo Fisher Scientific Inc., Rockford, IL, USA), 2mmol/L glutamine, and 1% penicillin-streptomycin-amphotericin (Lonza Walkersville, Inc Cells were maintained at 37 °C in a 5% CO_2_ incubator and serially subcultured to yield a morphologically homogeneous population of adherent fibroblasts under the microscopy and α-smooth muscle actin (SMA) immunohistochemical stain (Abcam, Cambridge, MA, USA) until the fourth passage. The cells were then stored at −170 °C. Fifth-passage fibroblasts (2.5 × 10^6^) were seeded in 1mL TCM in 10cm^2^ dishes. After reaching 90% confluence, the fibroblasts were washed twice with PBS (Thermo Fisher Scientific Inc.) and used for RNA. Total RNA was extracted using TRI reagent (Ambion, Carlsbad, CA, USA). The cell pellets were prepared in RIPA buffer for immunoblot analysis, and protein concentrations were measured using a BCA kit (Thermo Fisher Scientific Inc.).

### Transcriptome microarray and analysis of gene ontology and functional pathways

Total RNA was extracted from the fibroblasts and converted to cDNA, which was amplified and purified using an Illumina® Total Prep™ RNA Amplification Kit (Ambion, Carlsbad, CA, USA). A transcriptome assay was performed using a HumanHT-12 (BeadChip Illumina, San Diego, CA, USA) containing sequences representing ~47,315 probes, which covered 27,455 curated and putative genes. The quality and quantity of the extracted RNA were examined by a RNA quantification reagent (Ribogreen®, Invitrogen, Carlsbad, CA, USA). Fluorescence was determined using a fluorometer (Victor 3, Perkin Elmer, Boston, MA, USA). An IlluminaiScan scanner was used to create images of the microarrays. Intensities of the images were measured using GenomeStudio (v.2011.1, Illumina, Inc., San Diego, CA, USA) with Gene Expression Module (v1.0). The expression value of each gene was determined by calculating differences by perfect match intensity minus mismatch intensity of the probe pairs in use. Genes showing detection call *p*-values <0.01 were discarded to reduce the number of false positives, and the remaining 15,020 genes were analyzed. Fold change of gene expression was calculated as follows: a mean of expression levels of IPF-fibroblasts divided by that of control fibroblasts if the levels were higher in the IPF than the controls. In cases of countertrend, fold change was determined by dividing the mean value of control group by that of the IPF group and presented as minus value. The microarray data were analyzed using ScoreGenes software package (http://compbio.cs.huji.ac.il/scoregenes/). The general approach to analysis has been previously described [20]. Correction for multiple testing was performed by calculating the false discovery rate, as previously described [20]. Genes were defined as being substantially changed if they had a *P*-value of less than 0.05 by *t*-test, and a threshold number of misclassifications (TNoM) score of 0 and a *t*-test with a *P* value of less than 0.05 and absolute fold change of greater than 2, as previously described [21]. A heat map of the differentially expressed genes was constructed using the GenomeStudio software. Gene ontology enrichment was performed by the Gene Ontology (GO) database using the WebGestalt (http://www.webgestalt.org/), which was based on hypergeometric distribution to show the overrepresented gene ontology categories (*p* < 0.05). *P*-value was calculated using BINOMDIST function on the basis of overrepresentation of gene ontology categories when compared to all genes on the chip. The online program Pathway-Express (Onto-Tools, Wayne State University, Detroit, MI, USA, http://vortex.cs.wayne.edu/Projects.html) was used to explore biologically relevant pathways impacted by a list of input genes. The gene expression data were deposited in the NCBI Gene Expression Omnibus (series accession number GSE71351; (http://www.ncbi.nlm.nih.gov/geo/query/acc.cgi?acc=GSE71351). Gene ontology and pathway predictions were performed using the Gene Ontology database (http://www.webgestalt.org/) and Pathway-Express software (Onto-Tools; http://vortex.cs.wayne.edu/Projects.html).

### RT-PCR and real-time PCR of *CCL8* mRNA

Total RNA purified using TRI reagent was treated with a Turbo DNA-Free™ Kit (Ambion). Total RNA (3 μg) suspended in diethylpyrocarbonate-treated water was heated at 65 °C for 5 min with 0.5 μg of oligodeoxythymidine and 10 mM dNTPs, and then cooled on ice. Amplification was performed for 30 cycles (5min at 94 °C, 30s at 94 °C, 30s at 60 °C, and 30s at 72 °C) with extension at 72 °C for 7min. The following primer sequences were used: CCL8: sense 5′-TGGAGAGCTACACAAGAATCACC-3′andantisense5′-TGGTCCAGATGCTTCATGGAA-3′; β-actin: sense 5′-GGACTTCGAGCAAGAGATGG-3′ and antisense 5′-AGCACTGTGTTGGCGTACAG-3′. PCR products were separated on a 1.0% agarose gel containing ethidium bromide in Tris-borate EDTA buffer at 100V for 40min and visualized under UV light. The *CCL8* band intensities were normalized to those of β-actin. Real-time PCR was performed using the StepOne^TM^ Real-Time PCR System (Applied Biosystems, Foster city, CA, USA). The PCR mixture (20μL) contained 1μg cDNA, 1μL 10pmol forward and reverse primers, and 10μL 2 × Power SYBR Green PCR Master Mix (Applied Biosystems). The reaction was carried out in a two-step procedure: denaturation at 95 °C for 15s and 60 °C for 1min, and melting at 95 °C for 15 s, 60 °C for 1min, and 95 °C for 15s. Data were analyzed by the 2^-ΔΔCT^ method [22], presented as the relative fold change after normalization to β-actin.

### Determination of *CCL8* protein levels

BAL was performed in the mostly involved segments of IPF on HRCT without any immunosuppressive therapies or in the right middle lobe of normal control subjects as previously described [2, 23–25]. Total cell count was done using a hemocytometer. Differential count of five hundred cells was performed on slides of BAL cells prepared by a cytocentrifuge and stained with Diff-Quik. Cell pellets were separated from supernatants using centrifugation (500G, 5 min), and the supernatants were stored −80 °C. *CCL8* protein concentrations in BAL fluids and plasma were measured in normal controls and IPF patients using an ELISA kit (Abnova, Taipei, Taiwan) according to the manufacturer’s recommendations. The lower limit of detection was 1.5pg/mL; values below this limit were regarded as 0 pg/mL. The coefficients of variance for inter- and intra-assays were less than 15%. *CCL8* protein concentrations were measured in BAL fluids and plasma using an ELISA kit (Abnova, Taipei, Taiwan), and normalized to total protein concentration.

### Double immunofluorescence staining of *CCL8* and α-smooth muscle actin 

Paraffin blocks of IPF and control lung tissues were cut into 4-μm-thick slices, deparaffinized, rehydrated, and stained using hematoxylin and eosin. The sections were incubated in Fc receptor blocker (InnovexBiosciences, Richmond, CA, USA) for 30 min, incubated in TBS with 5% BSA for 1 h to block non-specific binding, and then incubated with monoclonal anti–human *CCL8* antibody (1:100, Origene, Rockville, MD, USA) or polyclonal anti-human α-SMA antibody (1:200, Abcam, Cambridge, MA, USA) in 5% BSA overnight at 4 °C. After washing with 1 × TBS, the sections were incubated with secondary antibodies: FITC-conjugated goat anti-rabbit antibody (1:2000, Abcam) and PE-conjugated donkey anti-mouse antibody (1:2000, Abcam). Nuclei were counterstained with DAPI (Invitrogen, Carlsbad, CA, USA). Confocal laser scanning was performed using a microscope (LSM 510 META at 100× magnification) coupled to a Photometrics Coolsnap HQ camera (Photometrics, Tucson, AZ, USA), and images were generated using the Zeiss LSM image browser.

### Statistical analysis

The data were analyzed using SPSS v. 20.0. The differences in gene expression between NC and IPF groups were considered statistically significant when the absolute fold-change in the mean value was >2 and the p-value was < 0.05 using *t*-tests and the nonparametric TNoM scoring method [21]. Comparisons of *CCL8* concentrations between the study groups were performed using the Kruskal-Wallis test and post hoc analysis (Mann-Whitney U test). A receiver operating characteristic (ROC) curve, AUC, and cut-off value were calculated using MedCalc [26]. Correlations between the *CCL8* levels and other parameters were analyzed by Spearman’s correlation coefficient. The data were presented as median values with 25 and 75% quartiles for skewed variables, or as the means ± SEM for those with a normal distribution. An optimal cutoff level of *CCL8* was calculated using Cutoff Finder [27] and survival rates were estimated by Kaplan-Meier’s method and compared using log-rank test. Values of *p* < 0.05 were considered statistical significance.

## Results

### Clinical characteristics of the study groups

Lung fibroblasts were cultured from 14 IPF-lungs and 10 control lungs. Among them, 8 IPF-fibroblasts and 4 controls were used for the transcriptome study (Additional file 1: Table S1). BAL samples were obtained from patients with IPF (*n* = 86), NSIP (*n* = 22), hypersensitivity pneumonitis (*n* = 20) and sarcoidosis (*n* = 19) (Table 1). The patients with IPF had significantly higher values for total cell count and numbers of macrophages, neutrophils and eosinophils in the BAL fluid, and lower FVC and DLCO values compared with those of NC (*p* < 0.05). The IPF group was comprised of 34 surgical IPF and 52 clinical IPF patients. There were no significant differences in the clinical and physiological parameters between the two groups (Additional file 1: Table S2). NSIP, hypersensitivity pneumonitis and sarcoidosis groups also had significantly higher values for total cell count and numbers of macrophages, neutrophils and eosinophils in the BAL fluid, and lower FVC and DLCO values compared with those of NC(*p* < 0.05).

**Table 1:** Clinical characteristics of the study subjects who underwent broncholaveolar lavage

Items	Normal controls	IPF	NSIP	HP	Sarcoidosis
No.	41	86	22	20	19
Age (year)	55(35–72)	67(59–75)*	60.1(39–70)	51.3(28–70)^†^	43.3 (28–69)^†^
Sex (male/female)	24/17	51/ 35	9/13	10/10	10/9
Smoke (CS/ES/NS)	9/11/14	19/25/36	2/5/12	3/3/12	5/2/9
Survival/Death	ND	57/21	ND	ND	ND
Follow up duration (years)	ND	3.6(1.6–6.5)	ND	ND	ND
FVC (% pred.)	106.1(87.0–119.0)	67.0(52.0–80.0)*	78.0(66.0–91.8)*	64.5(57.0–82.5)*	77.0(65.0–86.0)*
FEV1 (% pred.)	102.1(88.2–117.0)	83.0(59.0–93.0)*	85.0(73.8–101.3)*	74.5(64.3–92.0)*	85.0(64.0–101.0)*
DLCO (% pred.)	85.6(77.5–108.0)	57.0(46.0–71.0)*	76.0(59.0–92.0)*	67.0(55.0–90.0)*	75.5(57.8–84.5)*
dFVC (%/year)	NA	–7.1(–15.8–2.8)	NA	NA	NA
BAL total cell count (×10^4^/mL)	3.46 ± 0.82	7.58 ± 2.24*	17.64 ± 3.86*	13.03 ± 3.78*	8.45 ± 3.78*
Macrophages (×10^4^/mL)	3.02 ± 0.41	5.12 ± 1.71*	11.51 ± 3.07*	8.25 ± 2.38*	6.76 ± 3.79*
Neutrophils (×10^4^/mL)	0.21 ± 0.047	1.45 ± 0.38*	2.31 ± 1.01*	3.14 ± 2.36*	0.45 ± 0.16*
Eosinophils (×10^4^/mL)	0.02 ± 0.01	0.29 ± 0.07*	0.45 ± 0.14*	0.41 ± 0.19*	0.11 ± 0.07
Lymphocytes (×10^4^/mL)	0.04 ± 0.01	0.14 ± 0.05	2.68 ± 0.18*^†^	2.20 ± 0.19*^†^	2.17 ± 0.24*^†^

IPF: Idiopathic pulmonary fibrosis, NSIP: Nonspecific interstitial fibrosis, HP: Hypersensitivity pneumonitis

CS /ES/NS: current-smokers/ex-smokers/ never-smokers, ND: not determined, dFVC(%): annual decline rate of FVC

Difference in patient characteristics and pulmonary function test, shown as median (IQR), among the controls, IPF, NSIP, HP and sarcoidosis groups were calculated with Kruskal-Wallis analysis of variance and Mann-Whitney U-test as post-hoc test

BAL cell numbers, shown as mean ± standard error of the mean, among the five groups were compared using one-way ANOVA analysis of variance with Tukey’s honestly significant difference test as post-hoc test

Significances: Compared with control: *P <0.05, compared with IPF: ^†^ *P* < 0.05

### Global gene expression profiling of the IPF and control groups

**Fig. 1:**
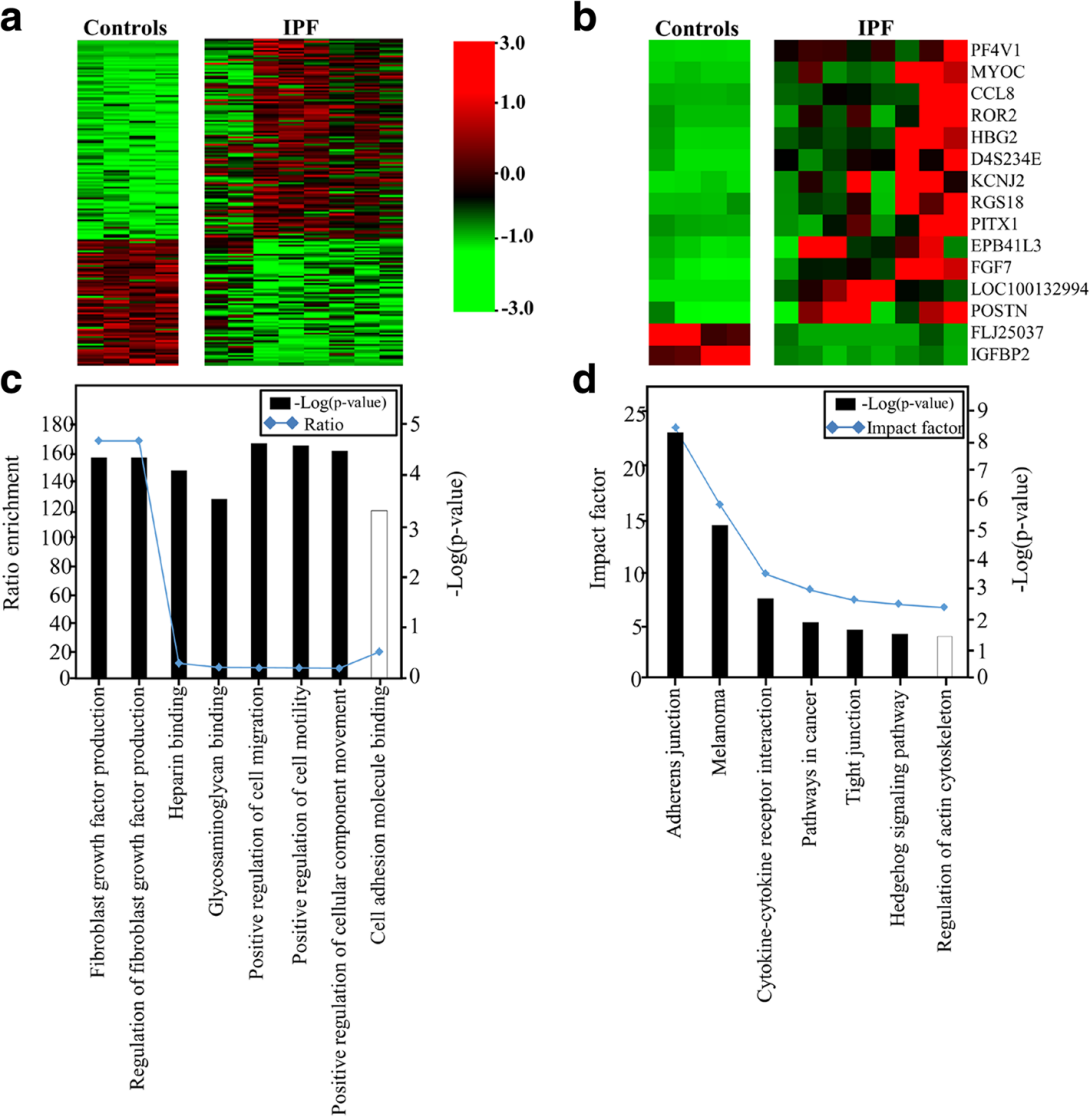
Gene expression profiles of fibroblasts derived from the lung tissues of 8 patients with IPF and 4 controls. **a** A heat map of 178 genes differentially expressed between the two groups (*p*-value <0.05 and absolute fold-change ratio >2 by *t*-test and TNoM). The maximum value (*red*) of each gene was set to 3, the minimum value to −3, and the remaining values were linearly fitted in the range. **b** A heat map of top 15 genes differentially expressed between the two groups (*p*-value < 0.05 by *t*-test and TNoM, absolute fold-change ratio >10). **c** The top 8 significantly perturbed Gene Ontology nodes in the IPF patients versus the controls. Left, statistical significance of the perturbation as determined by a gene set test; right, ratio of enrichment. The significance of differences between the dataset and the canonical pathway was measured as a ratio. Solid and open bars represent upregulation and downregulation, respectively. **d** Biological pathway analysis of differentially expressed gene sets related to IPF (corrected gamma *p*-value < 0.05). *P*-values and impact factors are plotted on the left and right axes, respectively

The cultured fibroblasts expressed α-SMA, but not E-cadherin on Western blotting (Additional file 2: Figure S1). The intensities of α-SMA were significantly higher in the fibroblasts from IPF lungs (*p* = 0.011). The expression levels of 15,020 genes were compared between the two groups. Unsupervised hierarchical clustering was done using the 15,020 genes in IPF fibroblasts and control fibroblast (Additional file 3: Figure S2). Although no gene passed the false discovery rate of less than 5% correction for multiple testing, 178 genes showed different expression levels according to the *t*-test and TNOM (*p* < 0.05 and fold changes > 2, Additional file 1: Table S3) (Fig. 1a). The expressions of 109 genes were increased, while that of 69 genes decreased in the IPF group compared to the control group. The top 15 genes showed 10-fold or greater changes: 13 genes (*PF4V1*, *MYOC*, *CCL8*, *ROR2*, *HBG2*, *D4S234E*, *KCNJ2*, *RGS18*, *PITX1*, *EPB41L3*, *FGF7*, *LOC*, and *POSTN*) were increased, and 2 genes (*FLJ25037* and *IGFBP2*) were decreased (Table 2 and Fig. 1b). *CCL8* expression was 22.8-fold higher in IPF-fibroblasts compared with control-fibroblasts.

**Table 2:** Genes showing 10-fold or greater changes in gene expression between the IPF and control groups

N	Gene Name	IPF (mean ± SE)	Controls (mean ± SE)	TNoM (*p*-value)	*t*-test (*p*-value)	FC
1	*PF4V1*	63.21 ± 18.03	1.07 ± 0.27	0.048	0.039	59.2
2	*MYOC*	18.5 ± 5.76	0.74 ± 0.24	0.048	0.018	25.1
3	*CCL8*	121.72 ± 45.48	5.33 ± 0.87	0.004	0.038	22.8
4	*ROR2*	70.83 ± 25.7	3.2 ± 2.7	0.048	0.034	22.1
5	*HBG2*	155.74 ± 48.34	8.07 ± 7.48	0.048	0.019	19.3
6	*D4S234E*	75.89 ± 21.97	4.04 ± 3.54	0.048	0.048	18.8
7	*KCNJ2*	111.29 ± 30.99	5.97 ± 2.12	0.004	0.011	18.6
8	*RGS18*	33.44 ± 12.08	1.99 ± 1.3	0.048	0.035	16.8
9	*PITX1*	592.66 ± 233.64	41.31 ± 14.57	0.048	0.05	14.3
10	*EPB41L3*	180.61 ± 52.92	12.91 ± 6.88	0.048	0.016	14.0
11	*FGF7*	31.28 ± 7.5	2.79 ± 1.17	0.004	0.007	11.2
12	*LOC100132994*	5.56 ± 1.38	0.5 ± 0.0	0.048	0.03	11.1
13	*POSTN*	88.86 ± 22.25	8.64 ± 7.85	0.048	0.009	10.3
14	*FLJ25037*	0.82 ± 0.21	8.79 ± 2.45	0.048	0.047	−10.8
15	*IGFBP2*	5.3 ± 1.18	60.29 ± 16.59	0.004	0.045	−11.4

Messenger RNA band intensity was quantified using GenomeStudio software (v. 2011.1; Illumina, Inc., San Diego, CA, USA) and the Gene Expression Module. Data were presented as the means ± standard error (SE). Gene expression fold-change was calculated as follows: if the mean level was higher in patients with IPF than in controls, the level in IPF fibroblasts was divided by that in control fibroblasts. In the reverse case (control higher than IPF levels), the mean value of the control group was divided by that of the IPF group and presented as a negative value. *P*-values were calculated using the nonparametric Threshold Number of Misclassifications (TNoM) scoring method and *t*-tests, and values less than 0.05 were considered significant

### Ontology and pathway analysis of the differentially expressed genes

A gene ontology analysis of the 178 genes was conducted. In comparison with those expected, the observed gene numbers were significantly higher in a total of 16 ontology categories (corrected *p*-values < 0.05; Additional file 1: Table S4). A ratio of enrichment of > 10 and a *p*-value < 0.001 was found in the following categories: regulation of fibroblast growth factor production (*p* = 4.6 × 10^−5^, ratio = 167.6) and cell adhesion molecular binding (*p* = 0.0005, ratio = 19.2) (Fig. 1c). The differentially expressed genes had a significant impact on pathways. Among 109 up-regulated genes, 11genes (*BMP2, CCL26, CCL8, EPAS1, EPB41L3, FGF7, GAS1, LEF1, PF4V1, PRKG2* and*PTGS2*) were mapped to 7 significant pathways (corrected gamma *p*-value < 0.05), including Adherens junction, Melanoma, Cytokine-cytokine receptor interaction, Pathways in cancer, Hedgehog signaling pathway, Tight junction, and Long-term depression (Additional file 1: Table S5 and Fig. 1d). Among 68 down-regulated genes, 5 genes (*CADM1, ITGA10, LLGL2, NLGN1* and *PRKCB*) were also mapped to 5 significant pathways (Regulation of actin cytoskeleton, Tight junction, Long-term potentiation, Cell adhesion molecules (CAMs), MAPK signaling pathway). *CCL8* was included in the ontology categories of extracellular region, receptor binding, heparin binding, G-protein-coupled receptor binding, chemokine activity, carbohydrate derivative binding, and glycosaminoglycan binding and the cytokine-cytokine receptor interaction pathway.

### Quantitation of *CCL8* mRNA and protein levels in fibroblasts

**Fig. 2:**
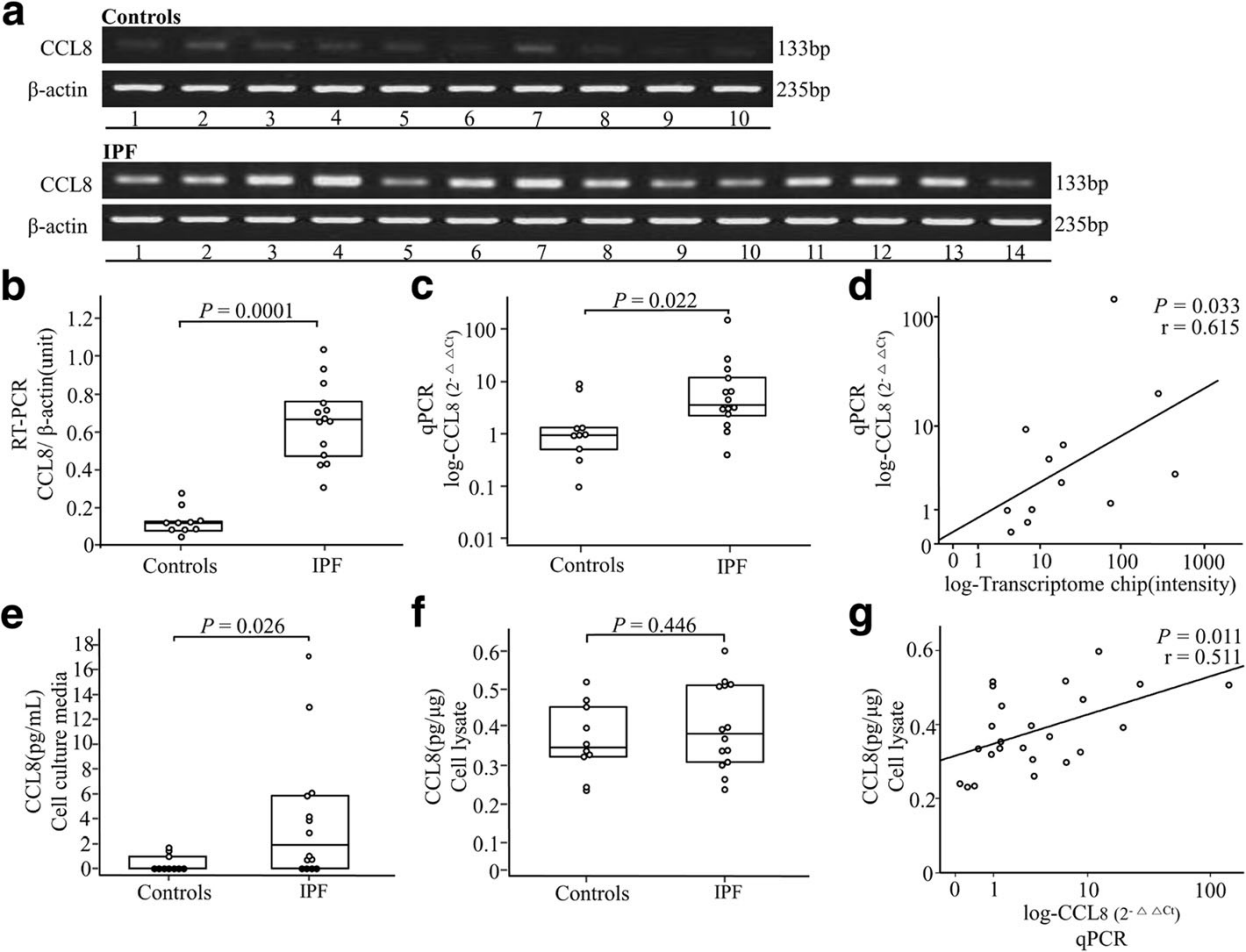
*CCL8* mRNA and protein levels in lung tissue-derived fibroblasts from 14 IPF patients and 10 controls. (**a**) RT-PCR, (**b**) densitometry of the *CCL8* RT-PCR band intensity normalized to that of β-actin, (**c**) real-time PCR, and (**d**) correlations of the *CCL8* mRNA levels of 12 subjects determined by the transcriptome chip with those by real-time PCR. **e**, **f** *CCL8* protein level of Culture media and cell lysate, and (**g**) correlations of the *CCL8* protein levels and *CCL8* mRNA levels of 24 subjects determined by the ELISA with those by real-time PCR. The data were presented as median values with 25 and 75% quartiles

Fibroblasts from 14 IPF lungs and 10 control lungs were used. The *CCL8* mRNA level normalized to that of *β-actin* was 3-fold higher in the IPF fibroblasts than in the control fibroblasts by RT-PCR (*p* = 0.0001; Fig. 2a and b) and real-time PCR (*p* = 0.022; Fig. 2c). The *CCL8* mRNA levels determined by the transcriptomic analysis showed a strong correlation to those determined by real-time PCR in the 12 subjects (r = 0.615, *p* = 0.033; Fig. 2d). In addition, we measured the *CCL8* protein amount in supernatants and cell lysates of the cultured fibroblasts (1 × 10^6^) using *CCL8* ELISA kit (Abnova, Taipei, Taiwan). *CCL8* levels were significantly higher in the supernatants from the IPF- fibroblasts compared those from the control-fibroblasts (*p* = 0.026) and cell lysates (*p* = 0.446) (Fig. 2e, f). The *CCL8* protein levels were well correlated with the *CCL8* mRNA levels (r = 0.511, *p* = 0.011) (Fig. 2g).

### *CCL8* protein levels in BAL fluids

**Fig. 3:**
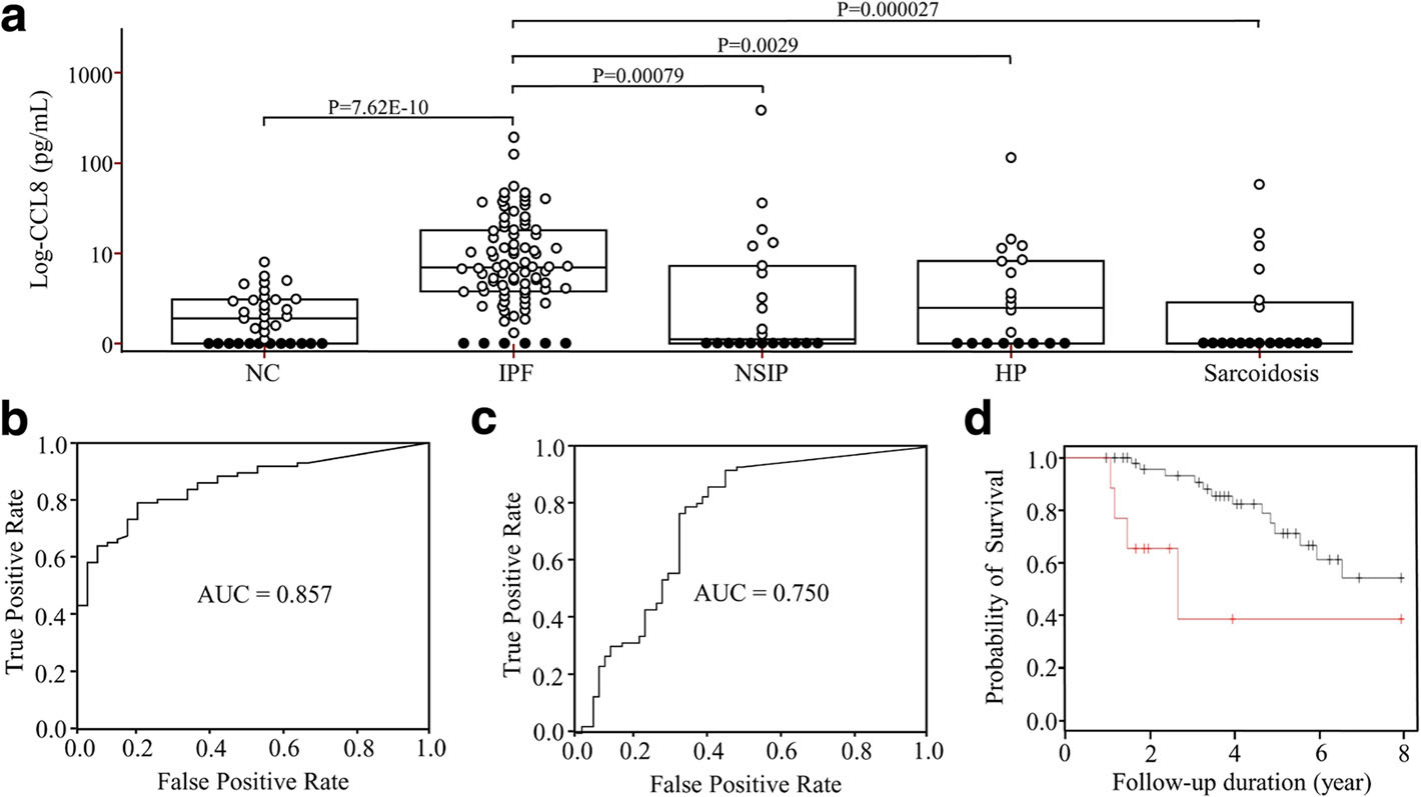
*CCL8* protein concentrations in BAL fluids and ROC curves. **a** *CCL8* protein was detected in 25 of 41 normal controls, 80 of 86 IPF patients, 11 of 22 NSIP patients, 8 of 20 HP patients and 13 of 19 sarcoidosis patients. Open and closed circles indicate *CCL8* protein levels detected (>1.5pg/mL) and those below the lower limit of detection, respectively. The data were presented as median values with 25 and 75% quartiles. **b** ROC curve of the *CCL8* protein concentration between the two groups. A cut-off value of 2.17pg/mL had 80.2% accuracy, 86.0% specificity, and 65.7% sensitivity for differentiating IPF patients from controls. **c** ROC curve of the *CCL8* protein concentration between the other interstitial lung diseases groups. A cut-off value 0.53pg/mL had 70.7% accuracy, 91.9% specificity, 57.4% sensitivity between the IPF patients and the other interstitial lung diseases group. **d** A Kaplan-Meier plot of 69 subjects with IPF followed up for 1to 8 years. The percent survival rate was markedly lower in the group with a *CCL8* level of >28.61 pg/mL (red line) compared with that in the group with a *CCL8* level of <28.61 pg/mL (black line) (hazard ratio = 3.93, CI: 1.25–12.39, *p* = 0.012)

The *CCL8* levels were significantly higher in the IPF patients than those in the NC [6.01(2.75–17.16pg/mL) vs. 0.90(0.00–2.07pg/mL), *p* = 7.62E-10], those in the NSIP [0.12(0.00–7.47pg/mL), *p* = 0.00079], those in the HP [1.52(0.00–7.37pg/mL), *p* = 0.0029] and those in the sarcoidosis patients [0.00(0.00–1.9pg/mL), *p* = 0.000027] (Fig. 3a). A ROC curve showed a clear difference between the IPF patients and NC (AUC = 0.857, Fig. 3b), and the other interstitial lung diseases groups (including NSIP, HP and sarcoidosis, *n* = 61) (AUC = 0.750, Fig. 3c). A cut-off value (2.29pg/mL) possessed 80.2% accuracy with 86.0% specificity and 65.7% sensitivity between the IPF patients and NC. A cut-off value (0.43pg/mL) possessed 70.7% accuracy with 91.9% specificity and 57.4% sensitivity between the IPF patients and the other interstitial lung diseases groups. The *CCL8* levels were analyzed in 69 subjects followed up for 1 to 8 years with respect to the survival rate. When the subjects were divided into two groups with a cut-off value of 28.61 pg/mL, the survival rate was significantly lower in the group > 28.61 pg/mL compared with that in the group < 28.61 pg/mL (hazard ratio = 3.93; CI: 1.25–12.39; *p* = 0.012; Fig. 3d). The *CCL8* protein concentrations showed a significant correlation with neutrophil (*p* = 0.014, r = 0.297), while no correlations with macrophage, lymphocytes and eosinophil numbers in BAL fluid and physiological parameters (Additional file 1: Table S6). There was no difference of plasma *CCL8* levels between 35 NC and 66 IPF patients (*p* = 0.167; Additional file 4: Figure S3A), and no correlation between *CCL8* concentrations in plasma and those in BAL fluids of 60 IPF patients (*p* = 0.169; Additional file 4: Figure S3B).

### Immunofluorescence staining for *CCL8*

**Fig. 4:**
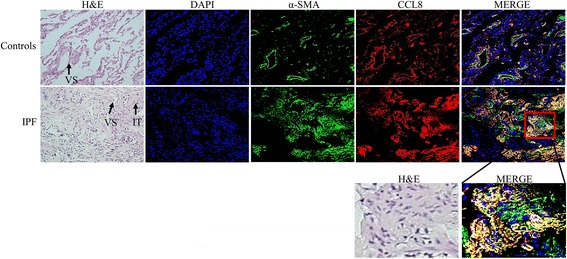
Representative double immunofluorescence-stained images of IPF and control lung tissues. *CCL8* and α-smooth muscle actin (α-SMA) were stained using PE- (red) and FITC-conjugated antibodies (green), respectively. A proportion of interstitial fibroblasts (IT) and the peribronchial and vascular area (VS) showed staining for both *CCL8* and α-SMA (magnification, 200×)

To confirm *CCL8* expression by myofibroblasts in IPF lungs, *CCL8* and α-SMA double immunofluorescence staining was performed in 3 IPF and 3 control lung tissues. In the control lungs, most probably smooth muscle cells were stained for both α-SMA and *CCL8*. In the IPF lungs, α-SMA was robustly expressed by interstitial fibroblasts, most but not all of which expressed *CCL8* (Fig. 4, Additional file 5: Figure S4).

## Discussion

In our study, 178 genes were found to be differentially expressed by the fibroblasts derived from IPF lungs. Among the top 15 genes of them, only *FGF7* and *POSTN* have been previously reported as related with IPF. The new genes identified in our study may provide insight into the pathogenesis of IPF. Among the top genes, *CCL8* expression was >20-fold higher in the IPF-fibroblasts. *CCL8* gene was reported to overexpress in 26 IPF lungs compared with 11 normal lungs (*p* = 0.099) in the GDS1252 dataset, as well as in 6 sarcoidosis lung compared with 6 normal lungs (*p* = 0.016) in the GDS3580 dataset of NCBI GEO DataSet Browser (https://www.ncbi.nlm.nih.gov/geo/). *CCL8* was included in the ontology categories of extracellular region, receptor binding, heparin binding, G-protein-coupled receptor binding, chemokine activity, carbohydrate derivative binding, and glycosaminoglycan binding, which are essential pathways in the pathogenesis of IPF. In validation, *CCL8* levels in BAL fluids appeared useful as a candidate marker for the differential diagnosis from NC and other chronic interstitial lung diseases. Thus, we identified several novel genes, and demonstrated the clinical relevance of *CCL8* as a candidate marker for the diagnosis and prognosis of IPF patients for the first time to the best of our knowledge.

*CCL8* is secreted into the peripheral circulation. However, no correlation between *CCL8* protein levels in BAL fluids and peripheral bloods was observed. This may be due to sources of *CCL8* other than fibroblasts or individual variance in the diffusion of *CCL8* from IPF-fibroblasts into the bloodstream. Among the CC chemokine ligand family, *CCL26* expression was increased in IPF patients compared with controls (2.39-fold increase, *p* = 0.048) in our study. In other studies of IPF-patients, *CCL2* are present in metaplastic epithelial cells and vascular endothelial cells [28] and *CCL3*, *CCL4*, and *CCL7* expressions are elevated in BAL fluids [29, 30]. The discrepancy between our data and these reports may be due to the presence of other sources of *CCL2*, *CCL3*, *CCL4*, and *CCL7* in the lung.

*CCL8* activates various immune cells, including mast cells, eosinophils, basophils, monocytes, T cells, and NK cells [31]. Recently, diverse functions of *CCL8* have been discovered in infection, immunity, and allergic inflammation. *CCL8* recruits gamma/delta T cells, which preferentially express *IL-17F* and synergistically enhance neutrophil chemotaxis in the presence of IL-8 [32]. *CCL8* is induced by fibroblasts and endothelial cells when co-stimulated with *IL1-β* and interferon *(IFN)-γ. IFN-γ* has also a synergistic effect with activation of *TLR2, TLR3* or *TLR4*. The application of both *IFN-γ* and dsRNA via TLRs resulted in the synergistic induction of *CCL8* expression [33]. All of them are known mediators involved in the development of IPF. Thus, *CCL8* appears to be related with innate immune response in the development of IPF, but the exact role of *CCL8* remains to be solved in near future.

In the ontology analysis of the 178 genes, regulation of fibroblast growth factor production, extracellular region, positive regulation of the EMT, and positive regulation of cell morphogenesis were the most relevant groups. The fibroblasts used in our study expresses markers of smooth muscle differentiation, such as α-SMA [34]. Lindahl GE and collaborators performed Gene ontology analysis using 843 differentially expressed genes between IPF and scleroderma fibroblasts and normal control fibroblasts. Enriched functional groups represent 12 broad categories as follows: anatomical structural development, regulation of cell cycle, response to stress and wounding, regulation of apoptosis, cell migration and smooth muscle contraction in upregulation and inflammatory and immune response, response to biotic stimulus, regulation of apoptosis, regulation of cell migration, regulation of cell proliferation, and regulation of I-kB/NF-kB cascade in down regulation [35]. The enriched functional groups are in part compatible with our findings. Studies using human whole lungs also showed elevated expression of genes related to tissue remodeling/reorganization and ECM formation/degradation [4, 5]. Interestingly, the expression of *CCL8* was positively correlated with those of *IL-8, IL-13RA2*, and *CCL2, ADAMTS1, ADAMTS8, MMP10, MMP2, MMP3, TIMP2, ECM1, TGFBI*, and *CLEC3B*, and inversely correlated with *FN1* (*p* < 0.05, respectively) (Additional file 1: Tables S7 and S8). However, the contents of the gene ontology are different between the studies. In our study, the following 18 genes were included in the extracellular region: *PF4V1*, *NID1*, *PTHLH*, *CREG1*, *TFPI*, *FBN2*, *RSPO3*, *TSKU*, *EPDR1*, *BMP2*, *CCL26*, *POSTN*, *MYOC*, *CCL8*, *C1QTNF9B*, *CLEC18C*, *FGF7*, and *TUBA4A.* Among them, *FGF7* [36], *POSTN* [37], *TSKU* [38], and *TFPI* [39] have been suggested to be involved in the pathogenesis of IPF. Two genes (*PTGS2* and *RGCC*) in our study were included in the fibroblast growth factor production and EMT categories. Among them, *PTGS2* is known to be involved in the development of IPF [40]. These results indicate that more than half of the genes in our study are newly identified.

Our study had the following limitation: the small number of lung tissue samples available for microarray analysis. Therefor we used the *t*-test and nonparametric TNoM scoring method to compare the differences in gene expression between NC and IPF groups because of no gene passed of less than 5% correction for multiple testing [21]. The use of unadjusted *P*-values may be less problematic than omitting informative genes in studies aimed at identifying target genes responsible for biological mechanisms [41]. In addition, when the differentially expressed genes in our study was compared with those with Lindahl GE’s study using cultured fibroblasts (*n* = 744, 10 controls and 3 IPF subjects) [35], those with Sridhr S’s study using cultured fibroblasts (*n* = 1813, 4 controls and 10 IPF subjects; GSE44723), those with Ronzani C’s study using (*n* = 3, 5 controls and 5 IPF subjects; GES45686) and those with Emblom-Callahan’s study using uncultured fibroblast (*n* = 1, 6 controls and 12 IPF subjects) [8]. Only 3–10% of the differentially expressed genes were overlapped between the studies as seen in a Venn diagram in the Supplement (Additional file 6: Figure S5). This discrepancy may be due to the small numbers of fibroblasts in each study in addition to the phenotypic changes of fibroblasts during the passage in culture.

Fibroblasts derived from IPF lungs have distinct biological characteristics: a high percentage of apoptotic cells, and increased collagen, fibronectin, gelatinase B, *TIMP*s, *β-FGF*, and *PDGF* expression (i.e., a pro-fibrotic secretory phenotype) [42], and a reduced capacity to secrete anti-fibrotic molecules, such as prostaglandin E2 and hepatocyte growth factor [9]. Interestingly, the above-mentioned genes were not identified in our study. Recently, a genomic expression in non-cultured fibroblasts obtained from IPF-lungs demonstrated 1,813 significantly differentially expressed transcripts from those of normal fibroblasts [8]. When they were compared with the 178 genes of our study, only 9 genes, including *ALDH3A2, CDC42EP3, IGFBP2, MOXD1, NBEAL2, PITX1, POSTN, TMEM51,* and *UBE2K,* were overlapped. This may be due to biological differences between the cultured fibroblasts used in our study and the uncultured ones from IPF-lungs. However, we validated the *CCL8* concentration in BAL fluid in 86 patients with IPF and those in 41 controls and *CCL8* protein expression using immunohistochemical stain with antibodies to *CCL8* and α-SMA-positive cells in the lung tissues of IPF. Because the BAL fluids and lung tissues were obtained in un-cultured condition, so *CCL8* protein may be generated per se, not solely due to a phenotypic shift of the fibroblasts. However, replication of the result is mandatory for useful biomarkers. Another limitation was the use of control fibroblasts from the resected cancer specimens. It cannot be excluded that the gene expression in fibroblasts derived from the lungs in which cancer has developed may be different form fibroblasts derived from the normal lungs.

Epigenetic changes, such as CpG methylation and miRNA, are widespread throughout the genome in IPF-lung tissues and may regulate the expression of important genes [43–46]. Although fibroblasts have been studied mainly at the steady-state RNA level, there is evidence for the abnormal regulation of mRNA translation in the fibroblasts [47]. Furthermore, the surrounding environment influences the gene expression of fibroblasts. Recently, diseased ECM was reported to be the predominant driver of pathological gene expression, and the expression of ECM-sensitive genes is regulated primarily at the translational level [48]. Genes encoding IPF-associated ECM proteins are targets for miR-29, which is downregulated in fibroblasts grown on IPF-derived ECM. Other candidate miRNAs are localized to the chromosome 14q32 microRNA cluster, many of which belong to the miR-154 family [49]. Thus, the differentially expressed genes in our study should be analyzed together with global changes in CpG methylation and miRNA expression in the future.

## Conclusion

Global gene expression profiling of fibroblasts from lung tissues from IPF patients was performed to identify novel candidate genes. *CCL8* was validated as a candidate in BAL fluids and lung tissues samples. A total of 178 genes showed differential expression; among 15 genes showing ≥ 10-fold changes, 13 were newly identified in relation with IPF. The *CCL8* protein concentrations in BAL fluids were significantly higher in patients with IPF, and a cut-off value of 2.29pg/mL showed a high degree of accuracy for diagnosis. The levels were also higher compared to those in other interstitial lung diseases including NSIP, HP and sarcoidosis with a cut-off value (0.43 pg/mL) possessing a high degree of accuracy for the discrimination from the other interstitial lung diseases. The subjects with IPF with *CCL8* levels >28.61 pg/mL showed a reduced survival rate. In conclusion, our transcriptome analysis identified new genes that may be involved in the pathogenesis of IPF. Among them, *CCL8* may be useful as a candidate molecule for the differential diagnosis of IPF and prediction of survival.

## Additional files

Additional file 1: Supplementary data. (DOC 379 kb)
Additional file 2: Figure S1. Expression of epithelial, mesenchyal and myofibroblast markers of IPF fibroblasts and controls. Fibroblasts, obtained from lung biopsies of 14 IPF and normal lung sections of 10 subjects with localized lung cancer, was characterized using Western blot analysis for E-cadherin (epithelial marker), vimentin (mesenchymal cell marker) and α-smooth muscle actin (myofibroblast marker). The expression levels were normalized to β-actin as an internal control protein. (TIF 8191 kb)
Additional file 3: Figure S2. A heat map of 15,020 genes in IPF fibroblast and control fibroblast. A gradient scale ranging between green (down-regulated) and red (up-regulated) was indicated. The maximum value (red) of each gene was set to 3, the minimum value to −3, and the remaining values were linearly fitted in the range. (TIF 18692 kb)
Additional file 4: Figure S3. Plasma *CCL8* concentrations in the study subjects and its correlation with *CCL8* levels in BAL fluids. (A) *CCL8* concentration in plasma from normal controls (*n* = 35) and IPF subjects (*n* = 66), and, (B) correlation of the paired samples between plasma and BAL fluids from IPF subjects (*n* = 60). The data were presented as median values with 25 and 75% quartiles. (TIF 3385 kb)
Additional file 5: Figure S4. A blocking study using a recombinant CCL8 protein in Immunofluorescence staining of CCL8 protein. The mouse-anti human CCL8 antibody (1:100) were incubated with 10, 1, 0.1ng of a recombinant CCL8 (Origene, Rockville, MD, USA) for 2 h at room temperature, then the mixtures were incubated for overnight with the tissue sections of IPF lung tissues at 4 °C. The 2nd Ab (rabbit anti-mouse-PE 1:2000) was incubated for 2 h and Confocal laser scanning was performed using a microscope. As shown in the pictures, intensity of CCL8 staining was decreased as the concentration of recombinant CCL8 protein increased. (TIF 8134 kb)
Additional file 6: Figure S5. Venn Diagram of the differentially expressed genes by 4 studies using the cultured and uncultured fibroblasts. The differentially expressed genes in our study (4 controls and 8 IPF-subjects, blue) were compared with those with Lindahl’s study using cultured fibroblasts (10 controls and 3 IPF subjects, yellow) [36], those with Sridhr’s study using cultured fibroblasts (4 controls and 10 IPF subjects, grey; GSE44723), those with Ronzani C’s study using cultured fibroblasts (5 controls and 5 IPF subjects, green; GSE45686) and those with Emblom-Callahan’s study using uncultured fibroblast (6 controls and 12 IPF subjects, red) [8]. (TIF 7160 kb) 

## Abbreviations

BAL: Bronchoalveolar lavage
ECM: Extracellular matrix
GO: Gene Ontology
HP: Hypersensitivity pneumonitis
IFN: Interferon
IPF: Idiopathic pulmonary fibrosis
NC: Normal controls
NSIP: Non-specific interstitial pneumonia
ROC: Receiver operating characteristic
