# Supplementary material for: Gene profile of fibroblasts identify relation of CCL8 with idiopathic pulmonary fibrosis
Source: Respir Res. 2017 Jan 5;18:3. doi: 10.1186/s12931-016-0493-6 (PMC5216573; doi:10.1186/s12931-016-0493-6)
Supplement: Additional file 1: — Supplementary data. (DOC 379 kb) [file 12931_2016_493_MOESM1_ESM.doc]

**Supplemental online data**

Title: Gene Profile of Fibroblasts identify relation of CCL8 with idiopathic pulmonary fibrosis

Authors:Jong-Uk Lee^1^, Hyun Sub Cheong^2^, Eun-Young Shim^1^, Da-Jeong Bae^1^, Hun Soo Chang^1,4^, Soo

-Taek Uh^5^, Young Hoon Kim^6^, Jong-Sook Park^4^, Bora Lee^7^, Hyoung Doo Shin^2,3^, Choon-Sik Park^1,4^

Supplementary Table 1. Clinical characteristics of the study subjects who underwent fibroblasts culture

| Items | Controls | IPF |
| --- | --- | --- |
| No. | 10 [4] | 14 [8] |
| Age (year) | 54(46-74) [54(48-59)] | 61(50-72) [64(50-72)] |
| Sex(male/female) | 3/7 [1/3] | 7/7 [4/4] |
| Smoke(CS/ES/NS) | 2/1/7 [0/0/4] | 4/4/6 [0/4/4] |
| Survival/Death | ND | 9/5 [5/3] |
| Follow up duration (years) | ND | 4.2(2.9-7.0) [4.1(2.1-7.0)] |
| FVC (% pred.) | 98(77-106) [98(87-106)] | 84(47-104) [84(56-99)]* |
| FEV1 (% pred.) | 103.3(98-118)[112(102-121)] | 94.9(81.8-108)[96(975.5-110)] |
| DLCO (% pred.) | 88(71-120) [90(76-120)] | 68(39-90) [60(39-87)] * |
| dFVC (%/year) | NA | -10(-32-13) [-17(-32-6)] |
| BAL total cell count (x10^4^/mL) | NA | 7.67±2.88 [6.85±2.71] |
| Macrophages (x10^4^/mL) | NA | 6.07±2.62 [4.71±2.38] |
| Neutrophils (x10^4^/mL) | NA | 0.29±0.13 [0.39±0.21] |
| Eosinophils (x10^4^/mL) | NA | 0.73±0.29 [0.81±0.55] |
| Lymphocytes (x10^4^/mL) | NA | 0.14±0.09 [0.24±0.17] |

Fibroblasts culture: Lung fibroblasts were cultured from normal lungs of 10 subjects (Controls) who underwent surgery to remove localized lung cancer and those from surgical biopsy specimens of 14 patients with IPF (IPF). The number in the parenthesis means characteristics of subjects, in whom transcriptome chip was performed.

CS /ES/NS: current-smokers/ex-smokers/ never-smokers, ND: not determined, dFVC(%): annual decline rate of FVC. Data are shown as median (IQR). Differences between the controls and the subjects with IPF were compared using Mann-Whitney U-test. Significances: Compared with control:* P <0.05

Supplementary Table 2. Clinical profiles of subjects with IPF subgroups

| Items | Surgical IPF | Clinical IPF |
| --- | --- | --- |
| No. | 32 | 54 |
| Age (year) | 67(59-75) | 68(59-74) |
| Sex (male/female) | 19/13 | 32/22 |
| Smoke (CS/ES/NS) | 9/10/11 | 10/15/25 |
| Survival/Death | 20/9 | 37/12 |
| Follow up duration (years) | 4.1(1.6-6.5) | 3.4(1.6-5.0) |
| FVC(% pred.) | 64(52-80) | 68(55-79) |
| FEV (% pred.) | 74(59-93) | 86(76-93) |
| DLCO(% pred.) | 59(50-72) | 56(40-70) |
| dFVC(%/year) | -12.4(-24.3 - -0.8) | -3.4 (-12.5 - 4) |
| BAL total cell count (x10^4^/mL) | 7.46±2.04 | 7.65±2.32 |
| Macrophages count (x10^4^/mL) | 5.02±1.53 | 5.16±1.42 |
| Neutrophils count (x10^4^/mL) | 1.43±0.58 | 1.51±0.33 |
| Eosinophils count (x10^4^/mL) | 0.27±0.12 | 0.29±0.17 |
| Lymphocytes count (x10^4^/mL) | 0.11±0.06 | 0.18±0.10 |

IPF was diagnosed by the presence of a UIP pattern in the pathological specimen (surgical IPF) and/or by HRCT in patients who were not subjected to surgical lung biopsy (clinical IPF).

CS /ES/NS:current-smokers/ex-smokers/never-smokers, dFVC(%): annual decline rate of FVC

Patient characteristics and pulmonary function test, shown as median (IQR), between the surgical IPF and clinical IPF were compared using Mann-Whitney U-test. BAL cell numbers, shown as mean ± standard error of the mean, between the two groups were compared using t-test. Significances: Compared with control:* P <0.05

Supplementary Table 3. A list of 178 differentially expressed genes by fibroblasts between IPF and controls

| N | Gene Name | IPF  (Intensity) | Controls (Intentsity) | TNOM  (p-value) | *t*-test  (p-value) | Fold change |
| --- | --- | --- | --- | --- | --- | --- |
| 1 | *PF4V1* | 63.21±18.03 | 1.07±0.27 | 0.048 | 0.039 | 59.2 |
| 2 | *MYOC* | 18.5±5.76 | 0.74±0.24 | 0.048 | 0.018 | 25.1 |
| 3 | *CCL8* | 121.72±45.48 | 5.33±0.87 | 0.004 | 0.038 | 22.8 |
| 4 | *ROR2* | 70.83±25.7 | 3.2±2.7 | 0.048 | 0.034 | 22.1 |
| 5 | *HBG2* | 155.74±48.34 | 8.07±7.48 | 0.048 | 0.019 | 19.3 |
| 6 | *D4S234E* | 75.89±21.97 | 4.04±3.54 | 0.048 | 0.048 | 18.8 |
| 7 | *KCNJ2* | 111.29±30.99 | 5.97±2.12 | 0.004 | 0.011 | 18.6 |
| 8 | *RGS18* | 33.44±12.08 | 1.99±1.3 | 0.048 | 0.035 | 16.8 |
| 9 | *PITX1* | 592.66±233.64 | 41.31±14.57 | 0.048 | 0.05 | 14.3 |
| 10 | *EPB41L3* | 180.61±52.92 | 12.91±6.88 | 0.048 | 0.016 | 14.0 |
| 11 | *FGF7* | 31.28±7.5 | 2.79±1.17 | 0.004 | 0.007 | 11.2 |
| 12 | *LOC100132994* | 5.56±1.38 | 0.5±0 | 0.048 | 0.03 | 11.1 |
| 13 | *POSTN* | 88.86±22.25 | 8.64±7.85 | 0.048 | 0.009 | 10.3 |
| 14 | *CLRN3* | 6.24±1.72 | 0.66±0.11 | 0.004 | 0.049 | 9.5 |
| 15 | *FBN2* | 1564.99±356.33 | 180.55±121.83 | 0.048 | 0.006 | 8.7 |
| 16 | *RASL12* | 199.94±67.69 | 24.78±24.13 | 0.048 | 0.039 | 8.1 |
| 17 | *DNER* | 22.83±7.74 | 2.83±1.54 | 0.048 | 0.037 | 8.1 |
| 18 | *GAS1* | 117.3±26.81 | 16.17±6.48 | 0.048 | 0.007 | 7.3 |
| 19 | *SEMA6A* | 43.6±12.18 | 6.14±3.27 | 0.048 | 0.018 | 7.1 |
| 20 | *FAM198B* | 89.99±21.27 | 12.71±10.7 | 0.048 | 0.035 | 7.1 |
| 21 | *LOC100129906* | 10.25±2.52 | 1.45±0.74 | 0.048 | 0.038 | 7.1 |
| 22 | *RSPO3* | 40.85±9.68 | 5.92±2.45 | 0.048 | 0.008 | 6.9 |
| 23 | *LOC286367* | 10.98±2.35 | 1.7±1.03 | 0.048 | 0.024 | 6.4 |
| 24 | *ATP8B4* | 115.81±36.76 | 18.84±8.41 | 0.048 | 0.034 | 6.1 |
| 25 | *MCC* | 9.06±2.15 | 1.52±0.59 | 0.048 | 0.037 | 6.0 |
| 26 | *HS.444999* | 9.72±1.85 | 1.66±0.69 | 0.048 | 0.014 | 5.9 |
| 27 | *C15ORF34* | 11.45±1.65 | 2.09±1 | 0.048 | 0.004 | 5.5 |
| 28 | *PTGS2* | 55.34±13.88 | 10.15±3.11 | 0.004 | 0.014 | 5.5 |
| 29 | *WDR52* | 5.85±0.94 | 1.09±0.59 | 0.048 | 0.007 | 5.4 |
| 30 | *C19ORF4* | 35.09±9.86 | 6.62±2.18 | 0.048 | 0.024 | 5.3 |
| 31 | *LOC93432* | 8.92±1.81 | 1.71±1.18 | 0.048 | 0.025 | 5.2 |
| 32 | *TSKU* | 194.24±43.46 | 37.35±18.54 | 0.048 | 0.009 | 5.2 |
| 33 | *CREG1* | 532.17±125.33 | 106.99±43.88 | 0.048 | 0.012 | 5.0 |
| 34 | *MYEF2* | 10.67±1.75 | 2.22±1.18 | 0.048 | 0.01 | 4.8 |
| 35 | *TRPA1* | 39.9±13.31 | 8.29±0.52 | 0.048 | 0.049 | 4.8 |
| 36 | *C14ORF68* | 11.29±1.14 | 2.45±1.95 | 0.048 | 0.002 | 4.6 |
| 37 | *FAM167A* | 207.12±43.34 | 45.67±11.66 | 0.048 | 0.029 | 4.5 |
| 38 | *FAM167A* | 207.12±43.34 | 45.67±11.66 | 0.048 | 0.03 | 4.5 |
| 39 | *HS.551145* | 55.28±13.4 | 12.73±2.76 | 0.048 | 0.015 | 4.3 |
| 40 | *LOC647954* | 9.31±1.32 | 2.26±1.02 | 0.004 | 0.006 | 4.1 |
| 41 | *BMP2* | 56.94±13.47 | 13.94±3.39 | 0.048 | 0.015 | 4.1 |
| 42 | *FAM167A* | 201.66±37.58 | 49.57±12.93 | 0.048 | 0.02 | 4.1 |
| 43 | *MOXD1* | 213.33±43.81 | 52.52±20.56 | 0.048 | 0.008 | 4.1 |
| 44 | *ARHGAP20* | 84.57±19.52 | 21.06±5.32 | 0.048 | 0.014 | 4.0 |
| 45 | *LOC400743* | 13.61±2.09 | 3.39±2.42 | 0.048 | 0.014 | 4.0 |
| 46 | *PRKG2* | 25.67±6.37 | 6.57±0.49 | 0.048 | 0.02 | 3.9 |
| 47 | *RDH10* | 847.32±179.47 | 222.46±49.97 | 0.048 | 0.01 | 3.8 |
| 48 | *LEF1* | 65.69±13.36 | 18.19±3.38 | 0.048 | 0.009 | 3.6 |
| 49 | *MME* | 108.6±18.74 | 30.1±12.31 | 0.048 | 0.02 | 3.6 |
| 50 | *GLRXP3* | 176.51±34.19 | 49.19±4.34 | 0.048 | 0.007 | 3.6 |
| 51 | *C1QTNF9B* | 32.43±5.87 | 9.46±1.8 | 0.004 | 0.005 | 3.4 |
| 52 | *TMEM51* | 489.68±93.82 | 148.45±23.09 | 0.048 | 0.008 | 3.3 |
| 53 | *LOC642443* | 13.15±1.72 | 4.06±2.4 | 0.048 | 0.012 | 3.2 |
| 54 | *HS.560319* | 9.86±1.91 | 3.07±1.49 | 0.048 | 0.044 | 3.2 |
| 55 | *PRR5* | 21.71±4.33 | 6.77±2.25 | 0.048 | 0.043 | 3.2 |
| 56 | *LOC100134466* | 11.1±1.82 | 3.46±2 | 0.048 | 0.027 | 3.2 |
| 57 | *HS.407903* | 113.74±19.06 | 35.94±5.61 | 0.004 | 0.019 | 3.2 |
| 58 | *CLEC18C* | 8.55±1.62 | 2.7±0.89 | 0.048 | 0.036 | 3.2 |
| 59 | *SDCBPP2* | 15.16±1.62 | 4.82±0.73 | 0.004 | 0.002 | 3.1 |
| 60 | *SLC25A45* | 11.12±0.83 | 3.6±1.42 | 0.004 | 0.001 | 3.1 |
| 61 | *TRPV4* | 5.57±0.95 | 1.82±0.67 | 0.048 | 0.027 | 3.1 |
| 62 | *HS.561415* | 18.48±2.3 | 6.07±1.89 | 0.004 | 0.006 | 3.0 |
| 63 | *ALDH3A2* | 44.58±9.99 | 14.75±1.4 | 0.048 | 0.02 | 3.0 |
| 64 | *S100A4* | 4854.82±637.15 | 1646.61±510.29 | 0.048 | 0.009 | 2.9 |
| 65 | *GYPE* | 6.37±0.77 | 2.17±0.68 | 0.048 | 0.006 | 2.9 |
| 66 | *ZNF98* | 8.45±1.72 | 2.9±0.39 | 0.004 | 0.015 | 2.9 |
| 67 | *EPDR1* | 657.57±78.58 | 227.14±42.65 | 0.004 | 0.001 | 2.9 |
| 68 | *KRT17* | 12.98±1.83 | 4.53±1.58 | 0.048 | 0.014 | 2.9 |
| 69 | *LOC100129104* | 71.1±15.04 | 25.24±1.44 | 0.048 | 0.019 | 2.8 |
| 70 | *TUBA4A* | 32.88±2.69 | 11.75±0.71 | 0.004 | 0.0003 | 2.8 |
| 71 | *SFT2D3* | 7.73±1.34 | 2.77±0.44 | 0.004 | 0.007 | 2.8 |
| 72 | *HS.145039* | 16.53±2.32 | 5.95±2.67 | 0.048 | 0.019 | 2.8 |
| 73 | *HS.542905* | 13.86±1.53 | 5±1.78 | 0.004 | 0.006 | 2.8 |
| 74 | *LOC442474* | 13.86±1.82 | 5.05±1.48 | 0.048 | 0.011 | 2.7 |
| 75 | *SNORD67* | 13.14±2.18 | 4.84±0.85 | 0.004 | 0.05 | 2.7 |
| 76 | *PTHLH* | 6.24±1.44 | 2.33±0.64 | 0.048 | 0.027 | 2.7 |
| 77 | *LOC729176* | 12.62±2.22 | 4.86±1.61 | 0.048 | 0.035 | 2.6 |
| 78 | *RGCC* | 1879.91±325.28 | 724.9±85.38 | 0.048 | 0.045 | 2.6 |
| 79 | *LOC729533* | 13.68±2.03 | 5.29±0.99 | 0.048 | 0.009 | 2.6 |
| 80 | *HS.200698* | 20.23±2.65 | 8.02±1.13 | 0.004 | 0.004 | 2.5 |
| 81 | *HS.560343* | 14.62±2.02 | 5.83±2.47 | 0.048 | 0.011 | 2.5 |
| 82 | *CABYR* | 58.13±9.19 | 23.19±3.44 | 0.048 | 0.026 | 2.5 |
| 83 | *SLC35D1* | 17.76±3.41 | 7.17±0.38 | 0.048 | 0.006 | 2.5 |
| 84 | *RNF144A* | 10.14±1.35 | 4.1±1.1 | 0.048 | 0.017 | 2.5 |
| 85 | *EPAS1* | 1615.97±241.09 | 654.28±127.68 | 0.048 | 0.016 | 2.5 |
| 86 | *SHANK3* | 34.38±5.46 | 14.04±4.04 | 0.048 | 0.023 | 2.4 |
| 87 | *LOC100129979* | 6.99±1.03 | 2.91±1.52 | 0.048 | 0.035 | 2.4 |
| 88 | *CCL26* | 24.69±2.25 | 10.32±2.19 | 0.048 | 0.048 | 2.4 |
| 89 | *C16orf74* | 33.95±3.13 | 14.34±1.4 | 0.048 | 0.002 | 2.4 |
| 90 | *ESPNL* | 92.5±15.38 | 39.66±6.23 | 0.048 | 0.002 | 2.3 |
| 91 | *TMTC1* | 203.63±26.03 | 88.47±9.6 | 0.048 | 0.011 | 2.3 |
| 92 | *HS.154513* | 18.95±2.15 | 8.25±2.98 | 0.048 | 0.013 | 2.3 |
| 93 | *NUDT8* | 14.48±1.28 | 6.32±2.69 | 0.048 | 0.016 | 2.3 |
| 94 | *ING5* | 5.04±0.8 | 2.21±0.37 | 0.048 | 0.01 | 2.3 |
| 95 | *FKBP1P1* | 11.56±1.6 | 5.12±1.56 | 0.048 | 0.038 | 2.3 |
| 96 | *LOC644929* | 8.39±1.24 | 3.75±1.15 | 0.048 | 0.03 | 2.2 |
| 97 | *HS.577021* | 12.24±1.02 | 5.51±1.32 | 0.004 | 0.039 | 2.2 |
| 98 | *TFPI* | 773.47±122.9 | 348.13±9.77 | 0.048 | 0.003 | 2.2 |
| 99 | *HS.584251* | 13.86±1.22 | 6.24±2.39 | 0.048 | 0.01 | 2.2 |
| 100 | *SIK2* | 20.99±3.3 | 9.62±0.96 | 0.048 | 0.01 | 2.2 |
| 101 | *HS.551074* | 15.01±2.05 | 6.89±2.06 | 0.048 | 0.01 | 2.2 |
| 102 | *HS.569162* | 29.27±4.46 | 13.54±1.14 | 0.048 | 0.033 | 2.2 |
| 103 | *UBE2K* | 13.08±1.5 | 6.07±3.28 | 0.048 | 0.036 | 2.2 |
| 104 | *KCTD12* | 66.25±13.34 | 31.38±1.96 | 0.004 | 0.047 | 2.1 |
| 105 | *AQP12B* | 12.68±1.26 | 6.02±1.28 | 0.048 | 0.035 | 2.1 |
| 106 | *NID1* | 70.52±10.09 | 34.22±5.05 | 0.048 | 0.008 | 2.1 |
| 107 | *CARD6* | 15.54±1.85 | 7.63±2.15 | 0.048 | 0.01 | 2.0 |
| 108 | *RBM47* | 16.5±2.12 | 8.12±2.14 | 0.048 | 0.027 | 2.0 |
| 109 | *DZIP1* | 19.54±1.41 | 9.63±2.11 | 0.004 | 0.033 | 2.0 |
| 110 | *PIGL* | 12.84±1.73 | 25.9±4.71 | 0.048 | 0.009 | -2.0 |
| 111 | *F2RL1* | 27.87±5.61 | 56.64±13.24 | 0.048 | 0.038 | -2.0 |
| 112 | *KLF2* | 545.01±120.45 | 1116.34±47.82 | 0.048 | 0.009 | -2.0 |
| 113 | *PBX4* | 10.57±2.3 | 21.82±1.53 | 0.004 | 0.009 | -2.1 |
| 114 | *DTNA* | 32.22±3.75 | 66.63±9.07 | 0.048 | 0.002 | -2.1 |
| 115 | *BATF3* | 20.92±5.43 | 43.94±2.1 | 0.048 | 0.016 | -2.1 |
| 116 | *SPDL1* | 106.9±23.66 | 225.71±59.99 | 0.048 | 0.048 | -2.1 |
| 117 | *NFE2L3* | 27.81±7.24 | 58.9±3.4 | 0.048 | 0.016 | -2.1 |
| 118 | *TRIM46* | 21.33±3.18 | 45.52±9.06 | 0.048 | 0.01 | -2.1 |
| 119 | *CEP152* | 13.22±3.38 | 28.34±4.02 | 0.048 | 0.022 | -2.1 |
| 120 | *HS.370423* | 7.15±1.35 | 15.57±2.42 | 0.048 | 0.008 | -2.2 |
| 121 | *SNORA77* | 21.6±4.14 | 47.19±6.4 | 0.048 | 0.006 | -2.2 |
| 122 | *RBP2* | 5.97±1.11 | 13.14±1.2 | 0.048 | 0.003 | -2.2 |
| 123 | *TDRD3* | 9.33±1.32 | 20.8±3.32 | 0.004 | 0.003 | -2.2 |
| 124 | *C5orf30* | 40.46±8.17 | 90.37±25.55 | 0.048 | 0.038 | -2.2 |
| 125 | *PRKCB* | 2.93±0.84 | 6.58±1.25 | 0.048 | 0.034 | -2.2 |
| 126 | *FABP5P1* | 8.81±2.3 | 19.81±3.1 | 0.048 | 0.019 | -2.2 |
| 127 | *MAOB* | 23.04±4.59 | 52.22±5.29 | 0.004 | 0.003 | -2.3 |
| 128 | *LOC728715* | 7.47±1.55 | 17±2.88 | 0.048 | 0.009 | -2.3 |
| 129 | *SCARNA9* | 7.79±1.36 | 17.75±1.93 | 0.004 | 0.002 | -2.3 |
| 130 | *HS.578895* | 7.03±0.8 | 16.2±1.67 | 0.004 | 0.0001 | -2.3 |
| 131 | *CDC42EP3* | 76.41±13.89 | 176.14±41.85 | 0.048 | 0.016 | -2.3 |
| 132 | *XRRA1* | 7.34±1.76 | 17.01±4.38 | 0.048 | 0.032 | -2.3 |
| 133 | *RGAG1* | 6.29±2.21 | 14.69±1.14 | 0.048 | 0.029 | -2.3 |
| 134 | *TPD52L1* | 36.2±8.32 | 85.26±8.13 | 0.048 | 0.004 | -2.4 |
| 135 | *SCARA3* | 221.05±59.51 | 524.67±17.06 | 0.048 | 0.001 | -2.4 |
| 136 | *FAM30A* | 6.75±1.9 | 16.57±2.55 | 0.048 | 0.013 | -2.5 |
| 137 | *DHRS8* | 5.16±1.26 | 12.81±1.57 | 0.004 | 0.005 | -2.5 |
| 138 | *SUMO1* | 4.6±1.07 | 11.52±1.29 | 0.048 | 0.003 | -2.5 |
| 139 | *HS.554478* | 6.37±1.31 | 15.98±1.93 | 0.004 | 0.002 | -2.5 |
| 140 | *HS.127715* | 4.04±0.86 | 10.15±2.4 | 0.048 | 0.013 | -2.5 |
| 141 | *LOC728844* | 4.2±0.91 | 10.62±3.49 | 0.048 | 0.038 | -2.5 |
| 142 | *PRUNE2* | 10.55±2.52 | 26.77±6.1 | 0.048 | 0.014 | -2.5 |
| 143 | *CELSR2* | 16.3±4.25 | 41.86±4.17 | 0.048 | 0.004 | -2.6 |
| 144 | *FLJ25006* | 3.6±0.86 | 9.32±2.31 | 0.048 | 0.016 | -2.6 |
| 145 | *RGL3* | 4.4±1.49 | 11.63±1.91 | 0.048 | 0.016 | -2.6 |
| 146 | *NBEAL2* | 5.35±1.24 | 14.28±1.94 | 0.004 | 0.002 | -2.7 |
| 147 | *GTPBP2* | 6.63±1.25 | 18.12±4.89 | 0.048 | 0.012 | -2.7 |
| 148 | *FBXO43* | 3.77±0.88 | 10.37±0.51 | 0.004 | 0.001 | -2.7 |
| 149 | *LOC389791* | 6.14±1.28 | 17.08±2.81 | 0.048 | 0.002 | -2.8 |
| 150 | *HS.563667* | 4.48±1 | 12.54±2.21 | 0.004 | 0.003 | -2.8 |
| 151 | *KIAA1549L* | 7.59±2.59 | 21.27±4.68 | 0.048 | 0.019 | -2.8 |
| 152 | *C15ORF42* | 4.78±1.74 | 13.67±1.68 | 0.048 | 0.009 | -2.9 |
| 153 | *NLGN1* | 2.87±1.04 | 8.4±2.82 | 0.048 | 0.045 | -2.9 |
| 154 | *BCHE* | 51.31±14.92 | 151.8±18.13 | 0.048 | 0.002 | -3.0 |
| 155 | *CDH13* | 68.5±16.44 | 204.75±19.8 | 0.048 | 0.001 | -3.0 |
| 156 | *CLGN* | 2.03±0.68 | 6.11±1.43 | 0.048 | 0.014 | -3.0 |
| 157 | *SAPCD1* | 6.36±1.75 | 19.15±3.69 | 0.048 | 0.005 | -3.0 |
| 158 | *ANKRD1* | 7.99±2.15 | 24.47±7.56 | 0.048 | 0.02 | -3.1 |
| 159 | *HS.549989* | 9.26±2.12 | 29.08±10.27 | 0.048 | 0.025 | -3.1 |
| 160 | *HS.565411* | 3.36±0.84 | 10.57±1.9 | 0.048 | 0.002 | -3.1 |
| 161 | *KIAA0649* | 4.29±1.39 | 13.57±2.23 | 0.048 | 0.004 | -3.2 |
| 162 | *HS.212830* | 3.76±0.8 | 11.93±1.54 | 0.004 | 3.74E-5 | -3.2 |
| 163 | *PRDM8* | 246.94±73.72 | 791.22±198.64 | 0.048 | 0.01 | -3.2 |
| 164 | *CADM1* | 90.31±30.84 | 294.91±41.52 | 0.048 | 0.003 | -3.3 |
| 165 | *LOC100131372* | 3.05±0.81 | 10.09±3.26 | 0.048 | 0.018 | -3.3 |
| 166 | *GPR56* | 59.1±17.42 | 207.86±22.3 | 0.004 | 4.86E-5 | -3.5 |
| 167 | *KIAA1324L* | 4.46±1.62 | 16.21±5.6 | 0.048 | 0.024 | -3.6 |
| 168 | *PTX3* | 2.97±1.94 | 10.99±2.99 | 0.048 | 0.043 | -3.7 |
| 169 | *DACH2* | 6.92±2.25 | 28.69±7.75 | 0.048 | 0.005 | -4.1 |
| 170 | *TNFRSF6B* | 32.26±8.37 | 140.5±29.03 | 0.048 | 0.029 | -4.4 |
| 171 | *ITGA10* | 6.66±1.8 | 30.39±4.13 | 0.004 | 0.97E-5 | -4.6 |
| 172 | *HOMER2* | 2.21±1.24 | 10.3±1.64 | 0.048 | 0.003 | -4.7 |
| 173 | *ZNF747* | 2.73±0.9 | 12.97±2.14 | 0.004 | 3.58E-5 | -4.8 |
| 174 | *LLGL2* | 0.68±0.15 | 3.51±0.54 | 0.004 | 0.054E-5 | -5.2 |
| 175 | *TM6SF1* | 10.33±3.09 | 55.34±10.57 | 0.004 | 3.15E-5 | -5.4 |
| 176 | *LAMP5* | 27.68±14.64 | 191.46±52.4 | 0.048 | 0.003 | -6.9 |
| 177 | *FLJ25037* | 0.82±0.21 | 8.79±2.45 | 0.048 | 0.047 | -10.8 |
| 178 | *IGFBP2* | 5.3±1.18 | 60.29±16.59 | 0.004 | 0.045 | -11.4 |

Messenger RNA band intensity was quantified using GenomeStudio software (v.2011.1, Illumina, Inc., San Diego, CA, USA) and the Gene Expression Module. Data were presented as the means ± SE. Gene expression fold change(FC) was calculated as follows: if the level was higher in IPF than controls, the mean expression level in IPF fibroblasts was divided by that in control fibroblasts. In the reverse case (control higher than IPF levels), the mean value of the control group was divided by that of the IPF group and presented as a negative value. P-values were calculated using the *t*-test and the nonparametric Threshold Number of Misclassifications (TNoM) scoring method, and values less than 0.05 were considered significant.

Supplementary Table 4. Candidate enriched gene sets for the differentially expressed genes in IPF groups compared to control group

| Gene Expression | Category | Gene Ontology Category | Genes | Number of reference genes in the category | Observed gene number | Expected  gene number | Ratio of enrichment | Significance of enrichment (Pcorr<0.05) | Significance of  enrichment  (raw P-value) |
| --- | --- | --- | --- | --- | --- | --- | --- | --- | --- |
| Up-regulation | biological process | fibroblast growth factor production | *PTGS2, RGCC* | 3 | 2 | 0.01 | 167.66 | 0.03 | 4.6E-05 |
|  |  | regulation of fibroblast growth factor production | *PTGS2, RGCC* | 3 | 2 | 0.01 | 167.66 | 0.03 | 4.6E-05 |
|  |  | heparin binding | *FGF7, PF4V1, POSTN, RSPO3, CCL8* | 122 | 5 | 0.45 | 11.13 | 0.009 | 8.3E-05 |
|  |  | glycosaminoglycan binding | *FGF7, PF4V1, POSTN, RSPO3, CCL8* | 164 | 5 | 0.6 | 8.28 | 0.03 | 0.0003 |
|  |  | positive regulation of cell migration | *FGF7, BMP2, PTGS2, CCL26, LEF1, ROR2, SEMA6A* | 220 | 7 | 0.87 | 8 | 0.02 | 2.5E-05 |
|  |  | positive regulation of cell motility | *FGF7, BMP2, PTGS2,CCL26, LEF1, ROR2, SEMA6A* | 224 | 7 | 0.89 | 7.86 | 0.02 | 2.8E-05 |
|  |  | positive regulation of cellular component movement | *FGF7, BMP2, PTGS2, CCL26, LEF1, ROR2, SEMA6A* | 232 | 7 | 0.92 | 7.59 | 0.02 | 3.5E-05 |
|  |  | positive regulation of locomotion | *FGF7, BMP2, PTGS2, CCL26, LEF1, ROR2, SEMA6A* | 232 | 7 | 0.92 | 7.59 | 0.02 | 3.5E-05 |
|  |  | regulation of cell migration | *BMP2, PTGS2, CCL26, LEF1, ROR2, MCC, RGCC, FGF7, SEMA6A* | 385 | 9 | 1.53 | 5.88 | 0.01 | 1.8E-05 |
|  |  | regulation of cell motility | *BMP2, PTGS2, CCL26, LEF1, ROR2, MCC, RGCC, FGF7, SEMA6A* | 407 | 9 | 1.62 | 5.56 | 0.02 | 2.8E-05 |
|  |  | regulation of locomotion | *BMP2, PTGS2, CCL26, LEF1, ROR2, MCC, RGCC, FGF7, SEMA6A* | 437 | 9 | 1.74 | 5.18 | 0.03 | 5.0E-05 |
|  |  | regulation of cellular component movement | *BMP2, PTGS2, CCL26, LEF1, ROR2, MCC, RGCC, FGF7, SEMA6A* | 445 | 9 | 1.77 | 5.09 | 0.04 | 5.7E-05 |
|  | molecular function | receptor binding | *PF4V1, KRT17, BMP2, CCL26, LEF1, ROR2, PTHLH, S100A4, CCL8, FGF7, SHANK3, DNER, RSPO3, SEMA6A* | 1123 | 14 | 4.14 | 3.39 | 0.004 | 3.7E-05 |
|  |  | tissue development | *KRT17, BMP2, PTGS2, LEF1, POSTN, ROR2, PTHLH, S100A4, RDH10, RGCC, FGF7, FBN2, DNER, RSPO3, MYEF2, ALDH3A2* | 1377 | 16 | 5.48 | 2.92 | 0.04 | 5.6E-05 |
|  | cellular component | extracellular region | *PF4V1, NID1, PTHLH, CREG1, TFPI, FBN2, RSPO3, TSKU, EPDR1, BMP2, CCL26, POSTN, MYOC, CCL8, C1QTNF9B, CLEC18C, FGF7, TUBA4A* | 1879 | 18 | 7.61 | 2.37 | 0.02 | 0.0003 |
| Down-regulation | molecular function | cell adhesion molecule binding | *NLGN1, CADM1, CDH13* | 54 | 3 | 0.16 | 19.23 | 0.03 | 0.0005 |

Supplementary Table 5. Biological pathways analysis of differentially expressed genes

| Gene Expression | Pathway Name | #Genes in Pathway | #Input Genes in Pathway | #Pathway Genes on Chip | Genes | Impact Factor | corrected p-value | corrected gamma p-value |
| --- | --- | --- | --- | --- | --- | --- | --- | --- |
| Up-regulation | Adherens junction | 78 | 1 | 75 | *LEF1* | 18.5 | 0.27 | 1.9E-07 |
|  | Melanoma | 71 | 1 | 71 | *FGF7* | 12.7 | 0.26 | 4.3E-05 |
|  | Cytokine-cytokine receptor interaction | 263 | 4 | 259 | *BMP2, CCL26, CCL8, PF4V1* | 8.7 | 0.02 | 0.002 |
|  | Pathways in cancer | 330 | 5 | 328 | *BMP2, EPAS1, FGF7, LEF1, PTGS2* | 7.7 | 0.01 | 0.004 |
|  | Hedgehog signaling pathway | 57 | 2 | 57 | *BMP2, GAS1* | 6.0 | 0.02 | 0.02 |
|  | Tight junction | 135 | 1 | 131 | *EPB41L3* | 5.8 | 0.43 | 0.02 |
|  | Long-term depression | 75 | 1 | 73 | *PRKG2* | 5.7 | 0.27 | 0.02 |
| Down-regulation | Regulation of actin cytoskeleton | 217 | 1 | 207 | *ITGA10* | 10.5 | 0.43 | 3.0E-04 |
|  | Tight junction | 135 | 2 | 131 | *LLGL2, PRKCB* | 6.0 | 0.05 | 0.02 |
|  | Long-term potentiation | 73 | 1 | 72 | *PRKCB* | 5.7 | 0.18 | 0.02 |
|  | Cell adhesion molecules (CAMs) | 134 | 2 | 129 | *CADM1, NLGN1* | 5.1 | 0.05 | 0.04 |
|  | MAPK signaling pathway | 272 | 1 | 266 | *PRKCB* | 5.0 | 0.51 | 0.04 |

| Supplementary Table 6. Correlation of CCL8 levels with cell profiles in BAL fluids and with lung function profiles | | | |
| --- | --- | --- | --- |
| Correlations | N | Correlation Coefficient | P-value |
| Macrophage(count) | 68 | -0.080 | 0.517 |
| Lymphocyte(count) | 68 | -0.056 | 0.650 |
| Neutrophil(count) | 68 | 0.297 | 0.014 |
| Eosinophil(count) | 68 | -0.108 | 0.383 |
| FVC% | 86 | -0.019 | 0.865 |
| DLCO% | 75 | -0.013 | 0.913 |

Supplementary Table 7. Correlation of expression levels between CCL8 and other genes related with inflammation and immune response.

| Inflammation/immunity | Gene | Correlation coefficient | P-value |
| --- | --- | --- | --- |
| Molecules | *HLA-A* | 0.224 | 0.484 |
|  | *HLA-B* | -0.139 | 0.665 |
|  | *G6PD* | 0.552 | 0.063 |
|  | *VCAM1* | -0.035 | 0.914 |
|  | *TNFAIP1* | 0.007 | 0.983 |
|  | *OAS2* | 0.042 | 0.898 |
|  | *OAS3* | -0.349 | 0.265 |
|  | *ISG15* | 0.098 | 0.762 |
|  | *ICAM1* | -0.049 | 0.88 |
| Cytokine | *IL6* | 0.238 | 0.457 |
|  | ***IL8*** | **0.755** | **0.005** |
|  | *IL10* | 0.413 | 0.183 |
|  | ***IL13RA2*** | **0.776** | **0.003** |
|  | *IL1B* | 0.549 | 0.065 |
|  | *ILK* | -0.51 | 0.09 |
|  | *TIMP1* | -0.035 | 0.914 |
| Chemokine | ***CCL2*** | **0.706** | **0.010** |
|  | *CCL5* | 0.399 | 0.199 |
|  | *CCL11* | 0.528 | 0.078 |

Supplementary Table 8. Correlation of expression levels between CCL8 and other genes related with extracellular matrix remodelling.

| ECM remodeling | Gene | Correlation coefficient | P-value |
| --- | --- | --- | --- |
| Collagens  & ECM Structural Constituents | *COL12A1* | -0.406 | 0.191 |
|  | *COL14A1* | 0.49 | 0.106 |
|  | *COL15A1* | 0.497 | 0.101 |
|  | *COL16A1* | 0.105 | 0.746 |
|  | *COL1A1* | 0.165 | 0.609 |
|  | *COL4A2* | 0.147 | 0.649 |
|  | *COL5A1* | 0.287 | 0.366 |
|  | *COL6A1* | 0.182 | 0.572 |
|  | *COL6A2* | 0.406 | 0.191 |
|  | *COL7A1* | 0.252 | 0.43 |
|  | *COL8A1* | 0.224 | 0.484 |
|  | ***FN1*** | **-0.594** | **0.042** |
| ECM Proteases | ***ADAMTS1*** | **0.58** | **0.048** |
|  | ***ADAMTS8*** | **0.727** | **0.007** |
|  | *MMP1* | 0.343 | 0.276 |
|  | ***MMP10*** | **0.718** | **0.009** |
|  | *MMP11* | 0.434 | 0.159 |
|  | *MMP14* | -0.042 | 0.897 |
|  | ***MMP2*** | **0.622** | **0.031** |
|  | ***MMP3*** | **0.762** | **0.004** |
|  | *SPG7* | -0.035 | 0.914 |
|  | *TIMP1* | -0.035 | 0.914 |
| ECM Protease Inhibitors | *COL7A1* | 0.252 | 0.430 |
|  | *THBS1* | 0.399 | 0.199 |
|  | *TIMP1* | -0.035 | 0.914 |
|  | ***TIMP2*** | **0.594** | **0.042** |
|  | *TIMP3* | 0.531 | 0.075 |
| Other ECM Molecules | *VCAN* | 0.154 | 0.633 |
|  | *CTGF* | -0.371 | 0.236 |
|  | ***ECM1*** | **0.615** | **0.033** |
|  | ***TGFBI*** | **0.643** | **0.024** |
|  | *THBS2* | 0.497 | 0.101 |
|  | *THBS3* | 0.091 | 0.779 |
|  | ***CLEC3B*** | **0.524** | **0.08** |
|  | *TNC* | -0.098 | 0.762 |
