# Supplementary figures and images for: Gene profile of fibroblasts identify relation of CCL8 with idiopathic pulmonary fibrosis
Source: Respir Res. 2017 Jan 5;18:3. doi: 10.1186/s12931-016-0493-6 (PMC5216573; doi:10.1186/s12931-016-0493-6)

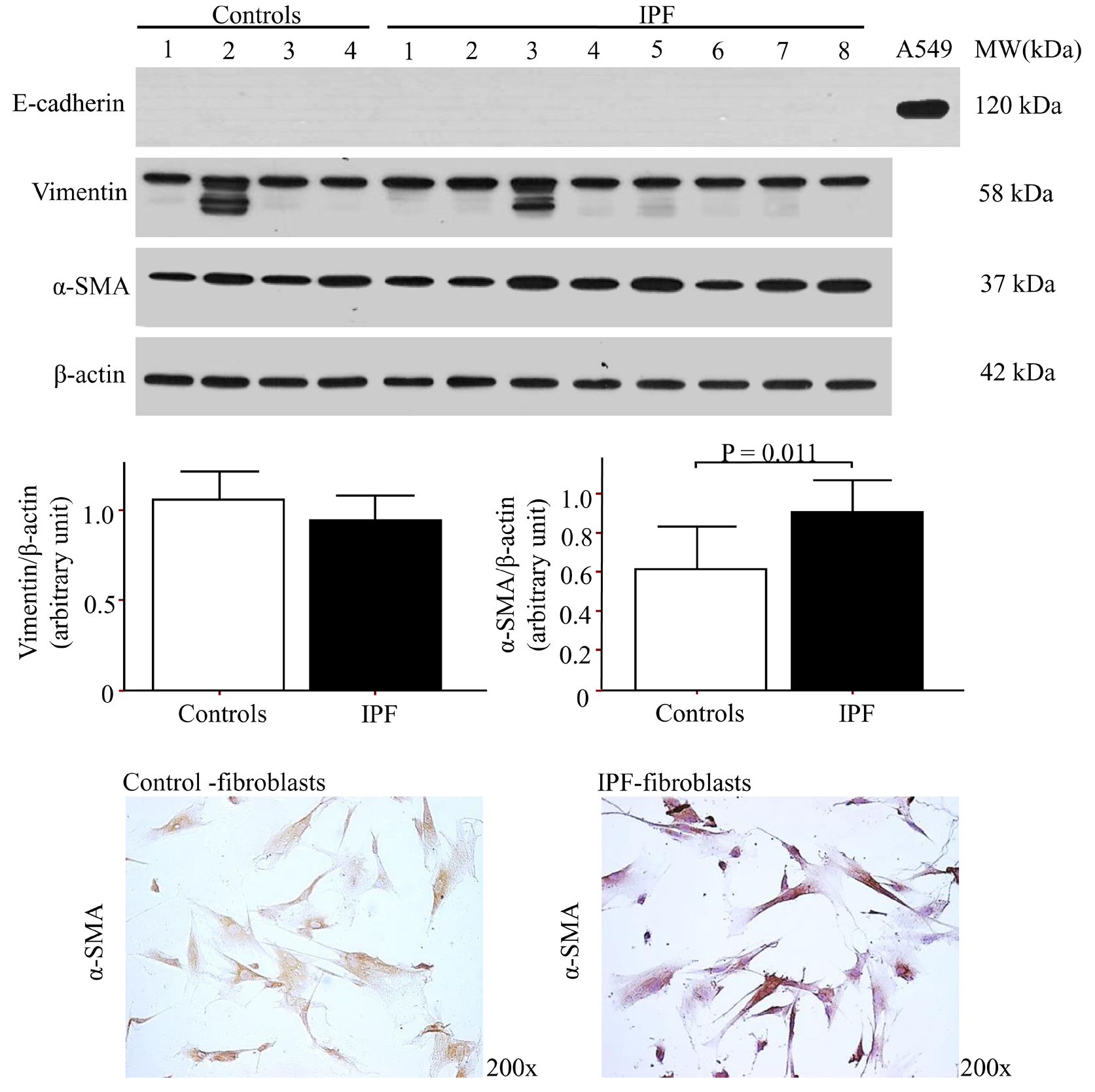

Supplement: Additional file 2: Figure S1. — Expression of epithelial, mesenchyal and myofibroblast markers of IPF fibroblasts and controls. Fibroblasts, obtained from lung biopsies of 14 IPF and normal lung sections of 10 subjects with localized lung cancer, was characterized using Western blot analysis for E-cadherin (epithelial marker), vimentin (mesenchymal cell marker) and α-smooth muscle actin (myofibroblast marker). The expression levels were normalized to β-actin as an internal control protein. (TIF 8191 kb) [file 12931_2016_493_MOESM2_ESM.tif]

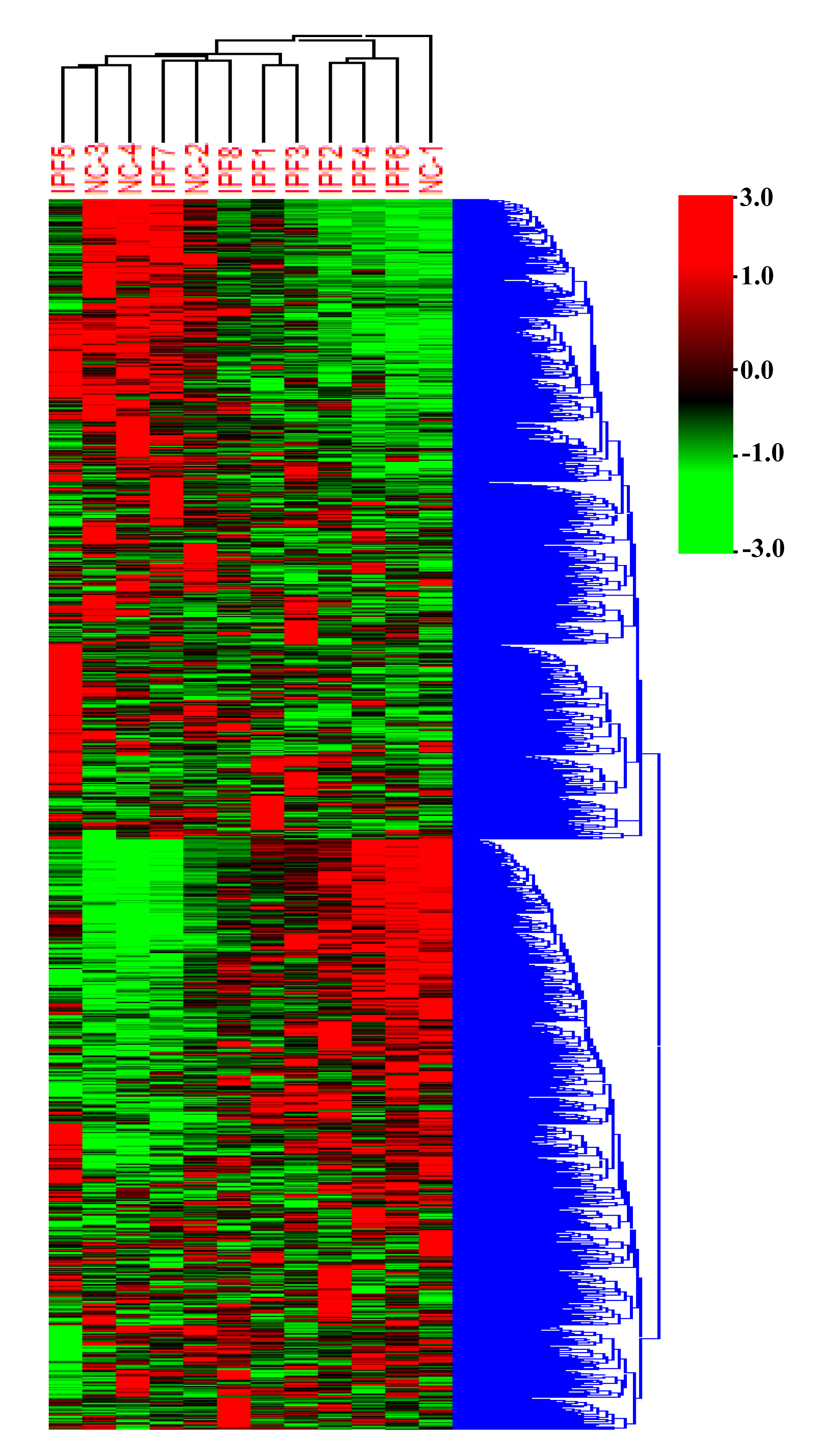

Supplement: Additional file 3: Figure S2. — A heat map of 15,020 genes in IPF fibroblast and control fibroblast. A gradient scale ranging between green (down-regulated) and red (up-regulated) was indicated. The maximum value (red) of each gene was set to 3, the minimum value to −3, and the remaining values were linearly fitted in the range. (TIF 18692 kb) [file 12931_2016_493_MOESM3_ESM.tif]

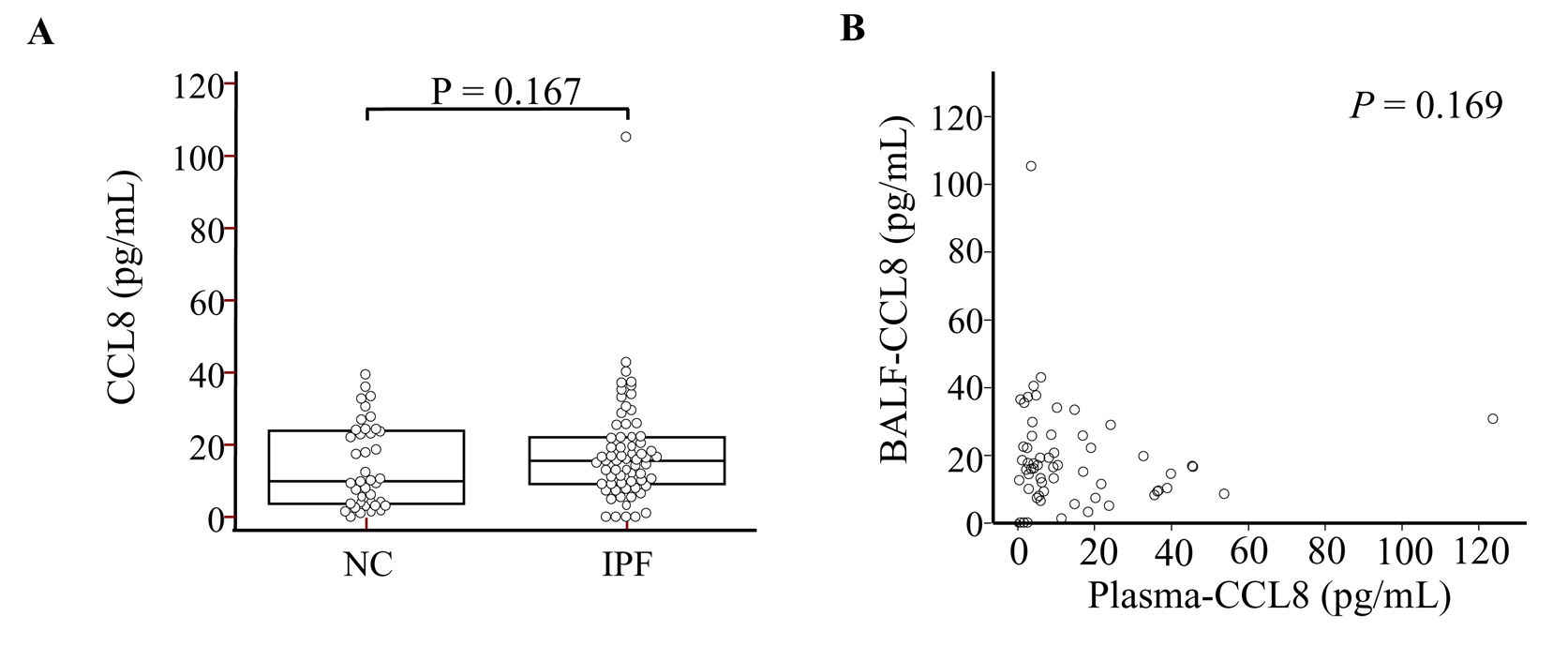

Supplement: Additional file 4: Figure S3. — Plasma CCL8 concentrations in the study subjects and its correlation with CCL8 levels in BAL fluids. (A) CCL8 concentration in plasma from normal controls (n = 35) and IPF subjects (n = 66), and, (B) correlation of the paired samples between plasma and BAL fluids from IPF subjects (n = 60). The data were presented as median values with 25 and 75% quartiles. (TIF 3385 kb) [file 12931_2016_493_MOESM4_ESM.tif]

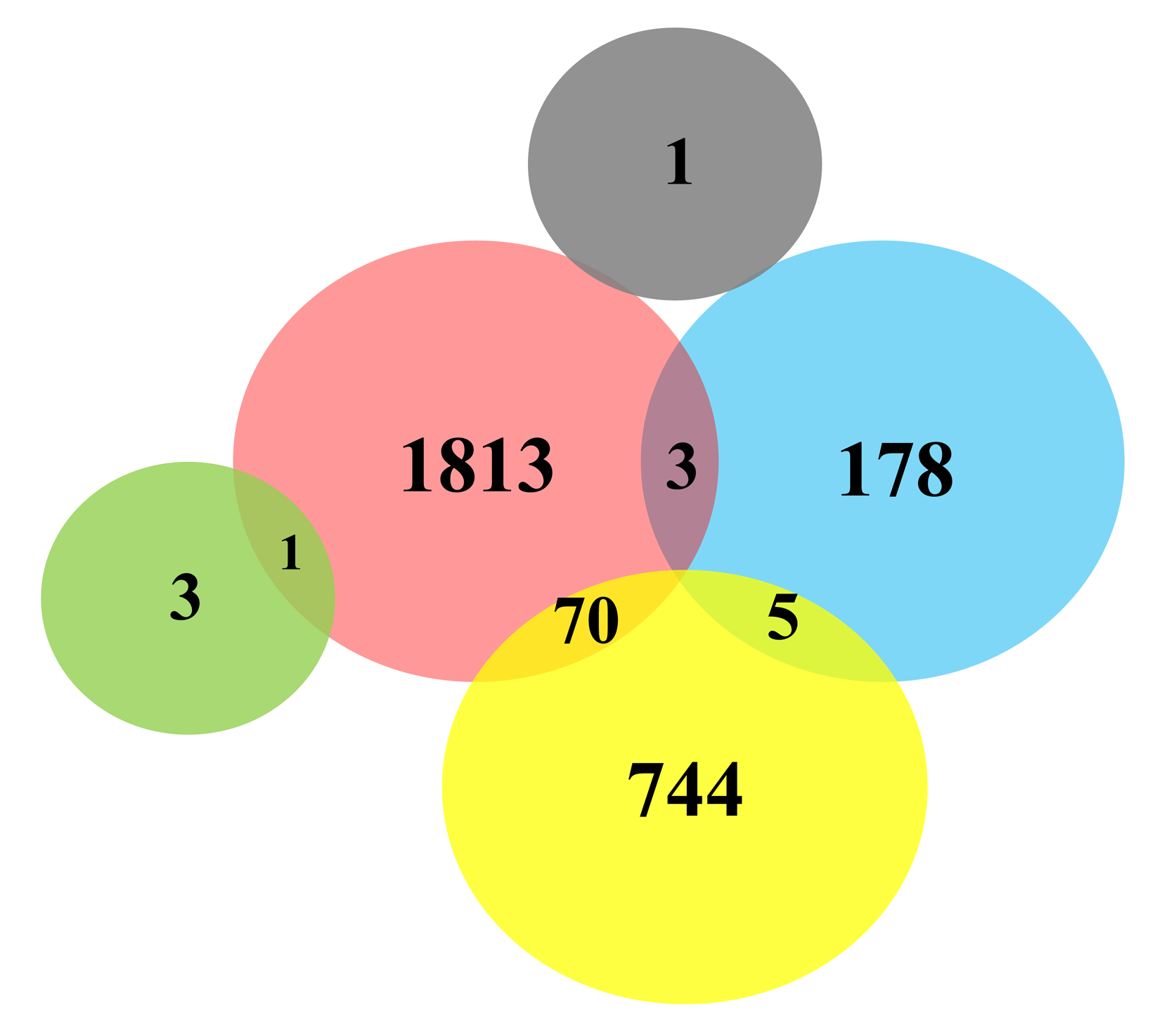

Supplement: Additional file 5: Figure S4. — A blocking study using a recombinant CCL8 protein in Immunofluorescence staining of CCL8 protein. The mouse-anti human CCL8 antibody (1:100) were incubated with 10, 1, 0.1ng of a recombinant CCL8 (Origene, Rockville, MD, USA) for 2 h at room temperature, then the mixtures were incubated for overnight with the tissue sections of IPF lung tissues at 4 °C. The 2nd Ab (rabbit anti-mouse-PE 1:2000) was incubated for 2 h and Confocal laser scanning was performed using a microscope. As shown in the pictures, intensity of CCL8 staining was decreased as the concentration of recombinant CCL8 protein increased. (TIF 8134 kb) [file 12931_2016_493_MOESM5_ESM.tif]

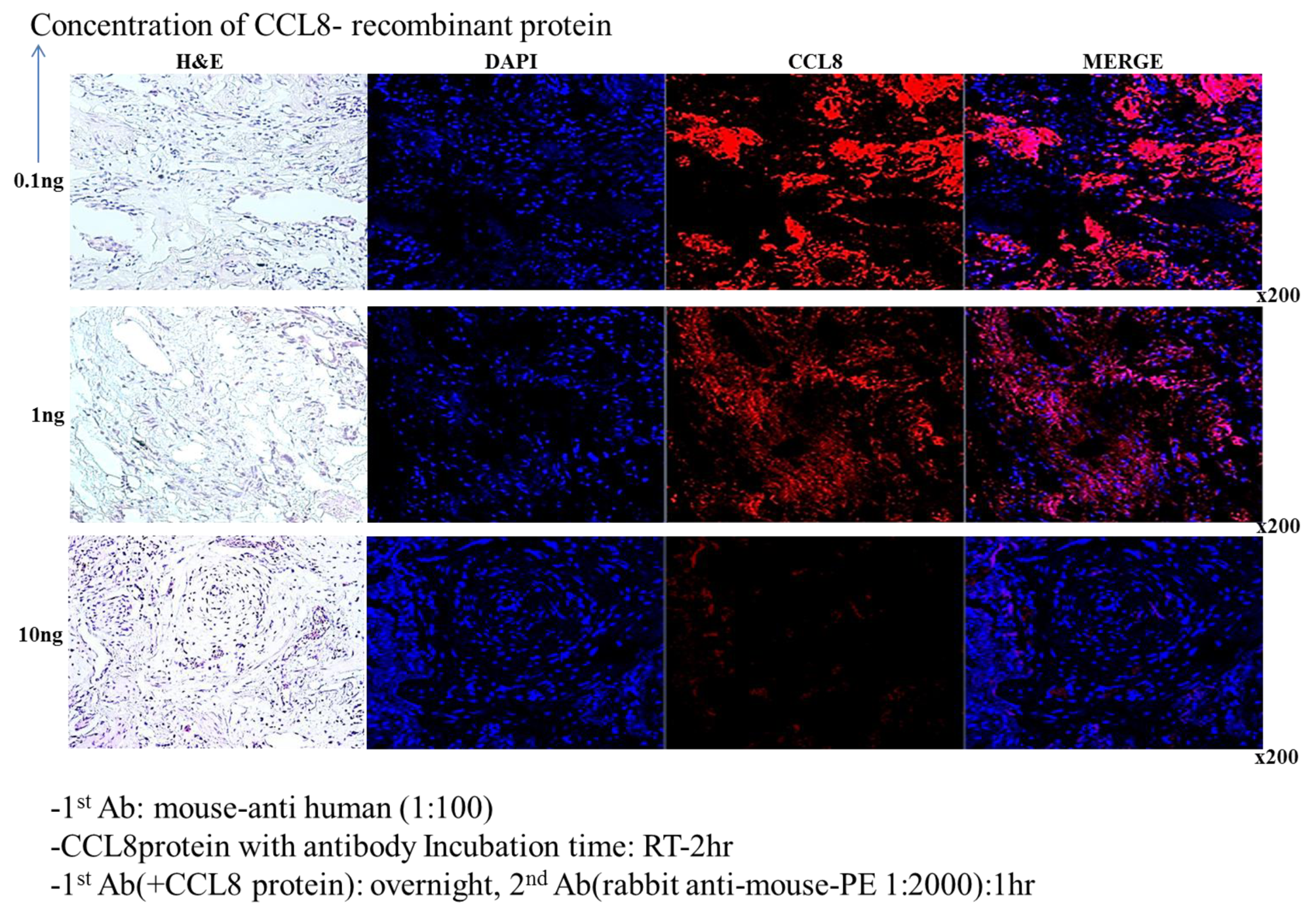

Supplement: Additional file 6: Figure S5. — Venn Diagram of the differentially expressed genes by 4 studies using the cultured and uncultured fibroblasts. The differentially expressed genes in our study (4 controls and 8 IPF-subjects, blue) were compared with those with Lindahl’s study using cultured fibroblasts (10 controls and 3 IPF subjects, yellow) [36], those with Sridhr’s study using cultured fibroblasts (4 controls and 10 IPF subjects, grey; GSE44723), those with Ronzani C’s study using cultured fibroblasts (5 controls and 5 IPF subjects, green; GSE45686) and those with Emblom-Callahan’s study using uncultured fibroblast (6 controls and 12 IPF subjects, red) [8]. (TIF 7160 kb) [file 12931_2016_493_MOESM6_ESM.tif]
